# Correlates of six-month housing instability among U.S. adults by veteran status: Exploratory study using data from the *All of Us* Program

**DOI:** 10.1371/journal.pone.0314339

**Published:** 2024-11-22

**Authors:** Hind A. Beydoun, Christian Mayno Vieytes, May A. Beydoun, Austin Lampros, Jack Tsai

**Affiliations:** 1 National Center on Homelessness among Veterans (NCHAV), Veterans Health Administration, Washington, DC, United States of America; 2 Department of Management, Policy, and Community Health, School of Public Health, University of Texas Health Science Center at Houston, Houston, TX, United States of America; 3 Laboratory of Epidemiology and Population Sciences, National Institute on Aging, NIA/NIH/IRP, Baltimore, MD, United States of America; 4 Department of Psychiatry, Yale School of Medicine, New Haven, CT, United States of America; Kansas City University, UNITED STATES OF AMERICA

## Abstract

Housing instability (HI) is a social determinant of health affecting adults in the United States (U.S.). Addressing HI among veterans is a national priority, and greater understanding of differences in HI between veteran and non-veteran populations would inform homeless services and research. We examined six-month prevalence and risk/protective factors associated with self-reported HI among veterans and non-veteran U.S. adults. Cross-sectional data from the *All of Us* Research Program (*AoU*) on 254,079 (24,545 veterans and 229,534 non-veterans) survey respondents were analyzed. Logistic regression models were constructed to examine rates of HI, and the association of HI with veteran status as well as demographic, socioeconomic, substance use, and health characteristics. Prevalence rates of HI were 14.9%, 11.5%, and 15.4%, in the general, veteran, and non-veteran populations, respectively. Veteran status was not significantly related to HI, after controlling for confounders. Male sex, middle age, unmarried status, lifetime cigarette smoking, and worse health were associated with greater HI odds, while higher income and health insurance availability were associated with lower HI odds, irrespective of veteran status. Racial disparities in HI were observed among non-veterans only. In addition, among non-veterans, adults who were unemployed or reported any lifetime alcohol consumption were more likely to experience HI, whereas any lifetime use of drugs was associated with lower likelihood of HI. In conclusion, although distinct sociodemographic and clinical correlates of HI were identified, HI did not differ by veteran status in a fully adjusted model.

## Introduction

Housing instability (HI) has been defined by researchers and practitioners as the state of living in housing but being at-risk of losing housing due to challenges such as difficulties paying rent, overcrowding, frequent relocation, and substantial proportion of income spent on housing [1,2]. U.S. veterans constitute a special population that may be at increased risk for HI because of detrimental experiences before, during, and after military service, although these risks may be partly mitigated by demographic and socioeconomic characteristics that favor veterans and the availability of health and social services through the U.S. Department of Veterans Affairs (VA) [3].

HI is an important social determinant of health affecting adults in the U.S. [4–7], and homelessness, defined as lack of stable, safe, and functioning housing, is a severe manifestation of HI [8,9]. In 2023, it was estimated that more than 600,000 individuals were homeless on a given night in the U.S. [10]. Among U.S. adults, the lifetime and 1-year prevalence rates of homelessness has been estimated at 4.2% and 1.5%, respectively [11], with veterans representing approximately 10% of the U.S. homeless population [6]. Tsai and Hooshyar analyzed data on HI among 1,004 low-income U.S. veterans from the 2021 National Veteran Homeless and Other Poverty Experiences study, and found that 10.9%, 8.0%, and 19.9% had lifetime histories of eviction, home foreclosure, and homelessness, respectively [7].

Homelessness prevention among U.S. veterans is a national priority [12]. HI has been identified as a key predictor of homelessness which, in turn, can adversely affect the health and social wellbeing of both veteran and non-veteran populations [7,13]. Yet, studies have rarely examined housing concerns by veteran status, and more studies are needed specifically on HI rather than homelessness per se [4]. Over the past few decades there have been major demographic changes within the U.S. veteran population [14] and various developments in housing and homeless services targeting both veterans and non-veteran populations [15–18]. Accordingly, up-to-date research is needed to elucidate differences in the burden of HI between veteran and non-veteran populations, and to identify risk/protective factors for HI that are specific to veteran and non-veteran populations for the purpose of homelessness prevention [19,20].

To date, a limited number of studies have examined differences in characteristics between homeless veteran and non-veteran populations in the U.S. [19,21], while others have identified high-risk groups for homelessness among U.S. veterans [22]. Recent studies focused on either veteran or non-veteran populations have identified a wide range of socio-demographic (*e*.*g*. sex, age, race/ethnicity, education, income, health insurance, foster care, entitlements, veteran status), behavioral (*e*.*g*. smoking status, alcohol consumption, substance use, sexual practices, sensation-seeking, aggression) and clinical (*e*.*g*. body mass index, chronic health conditions, physical/mental health) as correlates of homelessness [5–7,23].

Unlike homelessness, evidence pertaining to HI among U.S. veterans has been limited, with several studies focused on evaluating HI screening and intervention activities, and fewer of these studies focused on identifying risk/protective factors for HI [7,13,24–38]. Also, none of these studies have compared HI by veteran status among U.S. adults. In this exploratory study, we used data from the *All of Us* Research Program (*AoU*)–a unique database funded by the U.S. National Institute of Health–to examine the epidemiology of HI among U.S. veteran and non-veteran adult participants. We aimed to address the following research questions in our analyses: [1] which demographic, socioeconomic, substance use, and health characteristics previously linked to homelessness are associated with HI among veteran and non-veterans? and [2] Is veteran status associated with HI, net of other factors? Study findings are expected to contribute to the current understanding of HI epidemiology by veteran status.

## Materials and methods

### Data source

Initiated in 2018, the Precision Medicine *AoU* is a multidimensional, ongoing database that gathers information on a longitudinal cohort of U.S. adults from diverse backgrounds. Participants were recruited nationwide through convenience sampling as direct volunteers or through Healthcare Provider Organizations (HPOs), to facilitate studies of health disparities and those centered on communities traditionally underrepresented in biomedical research as described elsewhere[39–45]. As of December 27, 2023, more than 749,000 subjects have enrolled in the program, with more than 514,000 having completed its initial steps. The program has received more than 400,000 survey responses, more than 300,000 physical measurements, more than 400,000 electronic health records (EHR), and more than 500,000 bio-samples from participants aged 18 and older. The *AoU* was carried out in accordance with The Code of Ethics of the Declaration of Helsinki for experiments involving humans. Written informed consent was obtained from all participants ≥ 18 years of age prior to enrollment and procedures were performed in compliance with relevant institutional guidelines. The *AoU* Researcher Workbench employs a data passport model, through which authorized users do not need Institutional Review Board (IRB) review for each research project. Our project which used de-identified *AoU* data accessed and analyzed through the Researcher Workbench, a cloud-based platform for approved researchers, did not meet the definition of human subject research, and was exempt from IRB review. As such, written or verbal informed consent was waived by the *All of Us* IRB.

### Study population

**Fig 1:**
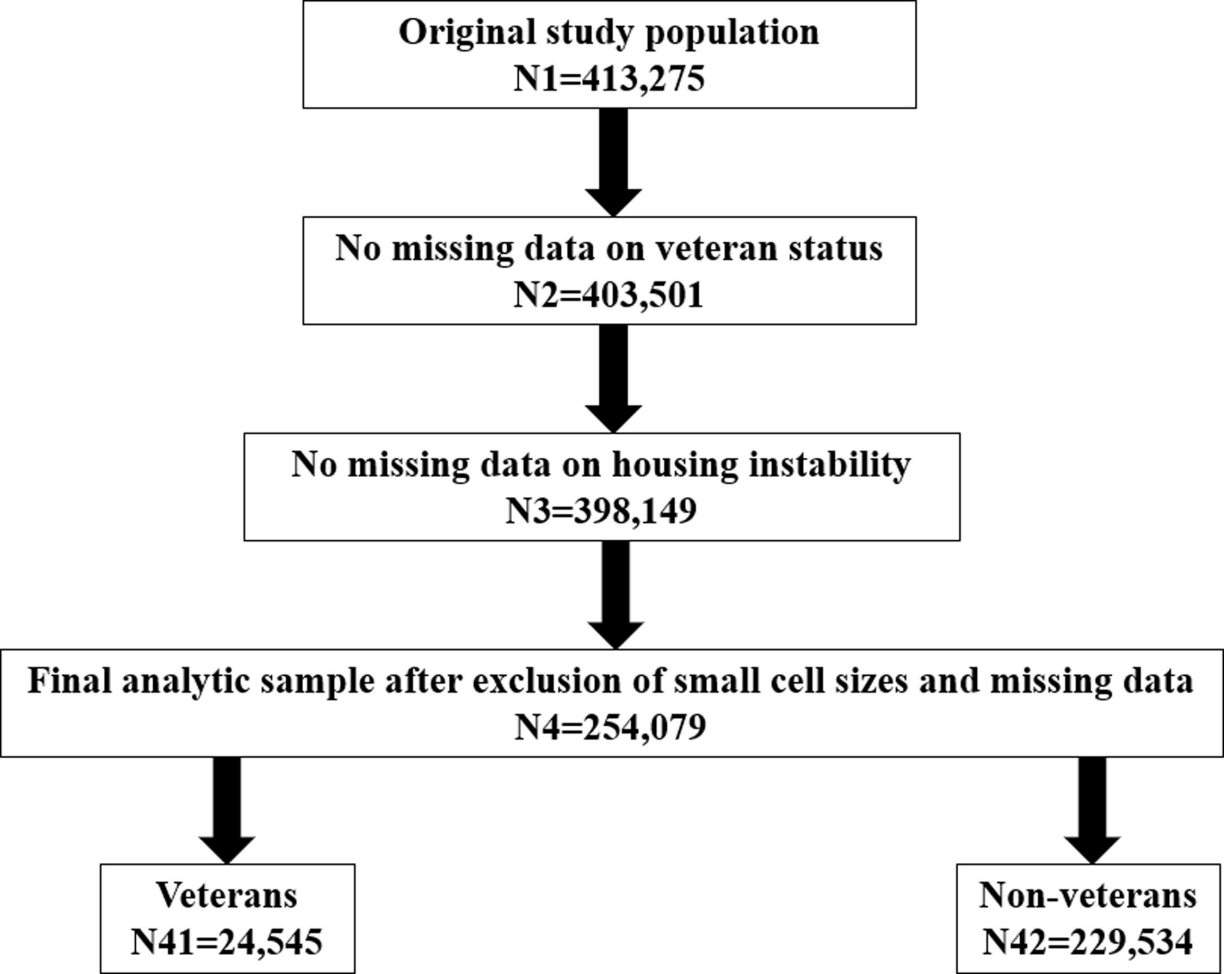
Study flowchart–*All of Us* research program.

Using the *AoU* Controlled Tier version 7 –the latest available version that allows access to specific data elements such as veteran status–we restricted the study to participants ≥ 18 years with no missing data on key variables. Of 413,275 participants ≥ 18 years of age, 403,501 had data on veteran status. After excluding 9,939 participants because they had missing data on the question pertaining to HI, a total of 398,149 participants were considered study eligible. Self-reported data were obtained on a subset of 397,191 participants who completed the three non-optional surveys, namely, “The Basics”, “Lifestyle”, and “Overall Health”, with a response rate of 99.8% among study-eligible participants enrolled in the *AoU* program. Due to small cell sizes, we restricted our sample to participants who reported their sex at birth as male or female, their race as White, Black, Asian, Other, Multiple/Unknown and their ethnicity as Hispanic/Latino, Not Hispanic/Latino, while excluding duplicate records and participants with missing data on the remaining demographic, socioeconomic, substance use, and health characteristics. As such, the final analytic sample consisted of 254,079 participants (61.7% female, age: 55.67±16.99 years, 65.3% white race), of which 24,545 were veterans and 229,534 were non-veterans (**Fig 1**). Significant differences were observed according to veteran status, HI, age, marital status, education, health insurance, tobacco use, and self-rated health, but not according to sex at birth, race, or ethnicity, between *AoU* participants included in and those excluded from complete case analyses. Percentages of subjects excluded from complete case analyses ranged between 32% and 36% for most of these characteristics, except for race/ethnicity whereby < 1% were excluded, suggesting that race and ethnicity were main contributors to missing data (**S1 Table**).

### Measures

#### Veteran status

Veterans and non-veterans were distinguished using the item “Have you ever served on active duty in the United States Armed forces, either in the regular military or in a National Guard or military reserve unit?” from “The Basics” survey component, with Yes or No being the available options through the “Controlled Tier” for data access.

#### Housing instability

Self-perception of HI over a six-months recall period was assessed using the item “In the past 6 months, have you been worried or concerned about not having a place to live?” from “The Basics” survey component, with Yes or No being available options, after exclusion of participants with missing data on this survey item.

#### Sociodemographic characteristics

Whereas age was obtained at screening, self-reported demographic data obtained from the “The Basics” survey component included sex (“What was your biological sex assigned at birth?”), and marital status (“What is your current marital status?”). In contrast, race and ethnicity were extracted from the “Person” domain of the *AoU* database. Socioeconomic data were obtained from the “The Basics” survey component using items for education (“What is the highest grade or year of school you completed?”), employment status (“What is your current employment status?”), income (“What is your annual household income from all sources?”), and health insurance (“Are you covered by health insurance or some other kind of health care plan?”). Whereas the employed category comprises those labeled employed for wages or self-employed, the unemployed category comprises those labeled as out of work, unable to work, retired, student, or homemaker.

#### Substance use and health characteristics

Using the “Lifestyle” survey component we assessed any lifetime history of substance use as dichotomous (Yes or No) variables for cigarette smoking (“Have you smoked at least 100 cigarettes in your entire life?”), alcohol consumption (“In your entire life, have you had at least 1 drink of any kind of alcohol, not counting small tastes or sips?”) and drug use (“In your lifetime, which of the following substances have you ever used?”), with drug types categorized as marijuana, hallucinogens, cocaine, methamphetamines, prescription stimulants, sedatives, prescription opioids, street opioids, inhalants, and other drugs. Notably, any lifetime use of a substance, in this context, included even a one-time use. Self-rated health (“In general, would you say your health is”) obtained from “Overall Health” survey component, was defined as Excellent, Very Good, Good, Fair or Poor.

### Statistical analysis

We performed data management and statistical analysis tasks on data from *AoU* participants using cohort and dataset creation tools provided on the Researcher Workbench, as well as statistical software, namely, R in Jupyter Notebook. A cohort was defined that meets the pre-specified eligibility criteria, whereas demographic, socioeconomic, substance use, and health characteristics were identified based on self-reported survey data. Bivariate associations were examined using independent samples t-tests for continuous variables, Pearson’s Chi-square tests for independence for categorical variables, or non-parametric counterparts, as appropriate. Unadjusted and adjusted odds ratios (OR) with their 95% confidence intervals (CI) were estimated using logistic regression models. First, multivariable logistic regression models were constructed to examine risk/protective factors associated with HI before and after stratifying by veteran status. Second, unadjusted and adjusted logistic regression models that sequentially controlled for demographic, socioeconomic, substance use, and health characteristics, were constructed to examine the relationship of veteran status with HI. Two-sided statistical tests were performed assuming an overall α level of 0.05.

## Results

The demographic, socioeconomic, substance use, and health characteristics of study participants are presented, overall, and by veteran status in **Table 1**. Compared to non-veterans, veterans were considerably older (67.9±14.8 years vs. 54.4±16.7 years), less frequently female (15.4% vs. 66.7%), Hispanic or Latino (6.6% vs. 15.3%), lacking health insurance (4.0% vs. 5.3%), or lacking any lifetime alcohol consumption (3.3% vs. 7.6%), but more likely to be out of work, unable to work, retired, students, or homemakers (66.7% vs. 46.7%) or to report cigarette smoking over their lifetime (56.6% vs. 38.1%). Nearly half of the population reported any lifetime use of drugs with no significant differences between veteran and non-veteran groups. Furthermore, there were significant differences in terms of the distributions by race, marital status, education, income, and self-rated health between veterans and non-veterans. Specifically, veterans were more likely to report being white, being ever married, having at least some college education, having an income that ranges between $35,000 and $150,000, as well as a ‘very good’ or ‘good’ self-rated health, than their non-veteran counterparts.

**Table 1:** Participant characteristics by veteran status–*All of Us* research program (n = 254,079)^a^.

	Total (n = 254,079)	Veteran status	
		Veteran (n = 24,545)	Non-Veteran (n = 229,534)
	N (%)	N (%)	N (%)
***DEMOGRAPHIC:***			
**Sex:**		P<0.0001	
**Female**	156,859 (61.7%)	3,784 (15.4%)	153,075 (66.7%)
**Male**	97,220 (38.3%)	20,761 (84.6%)	76,459 (33.3%)
**Age (years):**			
		P<0.0001	
**Mean ± SD**	55.67 ± 16.99	67.9 ± 14.8	54.4 ± 16.7
		P<0.0001	
**18–29**	16,438 (6.5%)	171 (0.7%)	16,267 (7.1%)
**30–39**	41,001 (16.1%)	1,225 (5.0%)	39,776 (17.3%)
**40–49**	38,832 (15.3%)	1,922 (7.8%)	36,910 (16.1%)
**50–59**	41,499 (16.3%)	2,882 (11.7%)	38,617 (16.8%)
**60–69**	52,516 (20.7%)	5,229 (21.3%)	47,287 (20.6%)
**70–79**	46,417 (18.3%)	7,993 (32.6%)	38,424 (16.7%)
**80+**	17,376 (6.8%)	5,123 (20.9%)	12,253 (5.3%)
**Race:**		P<0.0001	
**White**	165,986 (65.3%)	19,212 (78.3%)	146,774 (63.9%)
**Black**	41,637 (16.4%)	3,405 (13.9%)	38,232 (16.6%)
**Asian**	9,007 (3.5%)	261 (1.1%)	8,746 (3.8%)
**Other**	1,681 (0.7%)	83 (0.3%)	1,598 (0.7%)
**Multiple / Unknown**	35,768 (14.1%)	1,584 (6.4%)	34,184 (14.9%)
**Ethnicity:**		P<0.0001	
**Hispanic or Latino**	36,784 (14.5%)	1,615 (6.6%)	35,169 (15.3%)
**Not Hispanic or Latino**	217,295 (85.5%)	22,930 (93.4%)	194,365 (84.7%)
**Marital status:**		P<0.0001	
**Never married**	62,196 (24.5%)	2,782 (11.3%)	59,414 (25.9%)
**Married / Living with partner**	136,707 (53.8%)	15,456 (62.9%)	121,251 (52.8%)
**Separated / Divorced**	42,927 (16.9%)	4,858 (19.8%)	38,069 (16.6%)
**Widowed**	12,249 (4.8%)	1,449 (5.9%)	10,800 (4.7%)
***SOCIOECONOMIC:***			
**Education:**		P<0.0001	
**< High School**	15,670 (6.2%)	395 (1.6%)	15,275 (6.7%)
**High School / GED**	39,959 (15.7%)	3,388 (13.8%)	36,571 (15.9%)
**Some College**	66,249 (26.1%)	8,079 (32.9%)	58,170 (25.3%)
**College Graduate**	65,716 (25.9%)	6,169 (25.1%)	59,547 (25.9%)
**Advanced degree**	66,485 (26.2%)	6,514 (26.5%)	59,971 (26.1%)
**Employment status:**		P<0.0001	
**Employed**	130,576 (51.4%)	8,169 (33.3%)	122,407 (53.3%)
**Unemployed**	123,503 (48.6%)	16,376 (66.7%)	107,127 (46.7%)
**Income (U.S. dollars):**		P<0.0001	
**< 10,000**	36,790 (14.5%)	1,761 (7.2%)	35,029 (15.3%)
**10,000–25,000**	34,397 (13.5%)	2,857 (11.6%)	31,540 (13.7%)
**25,000–35,000**	22,208 (8.7%)	1,976 (8.0%)	20,232 (8.8%)
**35,000–50,000**	25,148 (9.9%)	2,953 (12.0%)	22,195 (9.7%)
**50,000–75,000**	34,846 (13.7%)	4,464 (18.2%)	30,382 (13.2%)
**75,000–100,000**	27,543 (10.8%)	3,358 (13.7%)	24,185 (10.5%)
**100,000–150,000**	34,427 (13.5%)	3,924 (15.9%)	30,503 (13.2%)
**150,000–200,000**	16,246 (6.4%)	1,554 (6.3%)	14,692 (6.4%)
**> 200,000**	22,474 (8.8%)	1,698 (6.9%)	20,776 (9.0%)
**Health insurance:**		P<0.0001	
**Yes**	240,875 (94.8%)	23,552 (96.0%)	217,323 (94.7%)
**No**	13,204 (5.2%)	993 (4.0%)	12,211 (5.3%)
***ANY LIFETIME SUBSTANCE USE:***			
**Tobacco use:**		P<0.0001	
**Yes**	101,326 (39.9%)	13,885 (56.6%)	87,441 (38.1%)
**No**	152,753 (60.1%)	10,660 (43.4%)	142,093 (61.9%)
**Alcohol consumption:**		P<0.0001	
**Yes**	235,887 (92.8%)	23,726 (96.7%)	212,161(92.4%)
**No**	18,192 (7.1%)	819 (3.3%)	17,373 (7.6%)
**Drug use:**		P = 0.065	
**Yes**	130,488 (51.3%)	12,743 (51.9%)	117,745 (51.3%)
**No**	123,591 (48.6%)	11,802 (48.0%)	111,789 (48.7%)
***HEALTH:***			
**Self-rated health:**		P<0.0001	
**Excellent**	30,779 (12.1%)	2,807 (11.4%)	27,972 (12.2%)
**Very good**	85,351 (33.6%)	8,799 (35.8%)	76,552 (33.3%)
**Good**	84,692 (33.3%)	8,226 (33.5%)	76,466 (33.3%)
**Fair**	42,967 (16.9%)	3,833 (15.6%)	39,134 (17.0%)
**Poor**	10,290 (4.0%)	880 (3.6%)	9,410 (4.1%)

*Abbreviations*: GED = General Education Development. ^a^ P values are based on Chi-square and independent samples t-tests, as appropriate. For employment status, the employed category comprises those labeled employed for wages or self-employed, and the unemployed category comprises those labeled as out of work, unable to work, retired, student, or homemaker.

Prevalence rates of self-perceived HI over the past 6 months among the overall, veteran, and non-veteran populations, were estimated to be 14.9%, 11.5%, and 15.4%, respectively. In addition, 7.4% of participants who reported HI were veterans. **Table 2** displays the bivariate relationships of HI with participant characteristics, overall, and by veteran status. HI was more prevalent among female veterans and male non-veterans. Also, HI was highest among Black participants and those between 40 and 59 years of age, irrespective of veteran status. Similarly, Hispanic/Latino participants had higher HI prevalence than their counterparts. High-risk groups for HI included participants who were never married, divorced, separated, or lacking health insurance. Among the overall, veteran, and non-veteran populations, higher income and having at least a college degree were inversely related to HI. Although unemployment was associated with a greater frequency of HI, the gap in HI was considerably less between employed and unemployed groups among veterans versus non-veterans. Whereas the 6-month prevalence of HI was lower among participants who had ever used alcohol or drugs, regardless of veteran status, participants who reported ever smoking cigarettes experienced HI at a higher rate than never-smokers. Although self-rated health was inversely related to HI, this association appeared to be stronger among non-veterans.

**Table 2:** Prevalence of self-perceived housing instability over the past 6 months by participant characteristics and veteran status–*All of Us* Research Program (n = 254,079) ^a^.

	Total (n = 254,079)	Veteran status	
		Veteran (n = 24,545)	Non-Veteran (n = 229,534)
	Yes/No [%]	Yes/No [%]	Yes / No [%]
		P<0.0001	
***TOTAL:***	38,069/216,010 [14.9%]	2,828/21,717 [11.5%]	35,241/194,293 [15.4%]
***DEMOGRAPHIC:***			
**Sex:**	P<0.0001	P<0.0001	P<0.0001
**Female**	21,736/135,123 [13.8%]	526/3,258 [13.9%]	21,210/131,865 [13.8%]
**Male**	16,333/80,887 [16.8%]	2,302/18,459 [12.5%]	14,031/62,428 [18.3%]
**Age (years):**			
	P<0.0001	P<0.0001	P<0.0001
**18–29**	2,931/13,507 [17.8%]	31/140 [18.1%]	2,900/13,367 [17.8%]
**30–39**	7,692/33,309 [18.8%]	268/957 [21.9%]	7,424/32,352 [18.7%]
**40–49**	7,907/30,925 [20.4%]	426/1,496 [22.2%]	7,481/29,429 [20.3%]
**50–59**	8,479/33,020 [20.4%]	621/2,261 [21.5%]	7,858/30,759 [20.3%]
**60–69**	7,839/44,677 [14.9%]	1,005/4,224 [19.2%]	6,834/40,453 [14.4%]
**70–79**	2,671/43,746 [5.8%]	364/7,629 [4.6%]	2,307/36,117 [6.0%]
**80+**	550/16,826 [3.2%]	113/5,010 [2.2%]	437/11,816 [3.5%]
**Race:**	P<0.0001	P<0.0001	P<0.0001
**White**	19,210/146,776 [11.6%]	1,604/17,608 [8.3%]	17,606/129,168 [11.9%]
**Black**	11,070/30,567 [26.5%]	886/2,519 [26.0%]	10,184/28,048 [26.6%]
**Asian**	744/8,263 [8.3%]	30/231 [11.5%]	714/8,032 [8.2%]
**Other**	236/1,445 [14.0%]	< 20 /66 [20.5%]	219/1,379 [13.7%]
**Multiple / Unknown**	6,809/28,959 [19.0%]	291/1,293 [18.4%]	6,518/27,666 [19.1%]
**Ethnicity:**	P<0.0001	P<0.0001	P<0.0001
**Hispanic or Latino**	7,129/29,655 [19.4%]	300/1,315 [18.6%]	6,829/28,340 [19.4%]
**Not Hispanic or Latino**	30,940/186,355 [14.2%]	2,528/20,402 [11.0%]	28,412/165,953 [14.6%]
**Marital status:**	P<0.0001	P<0.0001	P<0.0001
**Never married**	14,877/47,319 [23.9%]	731/2,051 [26.3%]	14,146/45,268 [23.8%]
**Married / Living with partner**	11,099/125,608 [8.1%]	805/1,4651 [5.2%]	10,294/110,957 [8.5%]
**Separated / Divorced**	10,542/32,385 [24.6%]	1,147/3,711 [23.6%]	9,395/28,674 [24.7%]
**Widowed**	1,551/10,698 [12.7%]	145/1,304 [10.0%]	1,406/9,394 [13.0%]
***SOCIOECONOMIC:***			
**Education:**	P<0.0001	P<0.0001	P<0.0001
**< High School**	4,921/10,749 [31.4%]	122/273 [30.9%]	4,799/10,476 [31.4%]
**High School / GED**	10,470/29,489 [26.2%]	753/2,635 [22.2%]	9,717/26,854 [26.6%]
**Some College**	13,069/53,180 [19.7%]	1,226/6,853 [15.2%]	11,843/46,327 [20.3%]
**College Graduate**	6,232/59,484 [9.5%]	455/5,714 [7.4%]	5,777/53,770 [9.7%]
**Advanced degree**	3,377/63,108 [5.1%]	272/6,242 [4.2%]	3,105/56,866 [5.2%]
**Employment status:**	P<0.0001	P = 0.0010	P<0.0001
**Employed**	15,020/115,556 [11.5%]	864/7,305 [10.6%]	14,156/108,251 [11.6%]
**Unemployed**	23,049/100,454 [18.7%]	1,964/14,412 [11.9%]	21,085/86,042 [19.7%]
**Income (U.S. dollars):**	P<0.0001	P<0.0001	P<0.0001
**< 10,000**	14,768/22,022 [40.1%]	866/895 [49.2%]	13,902/21,127 [39.7%]
**10,000–25,000**	9,452/24,945 [27.4%]	800/2,057 [28.0%]	8,652/22,888 [27.4%]
**25,000–35,000**	4,383/17,825 [19.7%]	301/1,675 [15.2%]	4,082/16,150 [20.2%]
**35,000–50,000**	3,509/21,639 [13.9%]	341/2,612 [11.5%]	3,168/19,027 [14.3%]
**50,000–75,000**	2,892/31,954 [8.3%]	278/4,186 [6.2%]	2,614/27,768 [8.6%]
**75,000–100,000**	1,373/26,170 [4.9%]	96/3,262 [2.9%]	1,277/22,908 [5.3%]
**100,000–150,000**	1,058/33,369 [3.1%]	100/3,824 [2.5%]	958/29,545 [3.1%]
**150,000–200,000**	333/15,913 [2.0%]	22/1,532 [1.4%]	311/14,381 [2.1%]
**> 200,000**	301/22,173 [1.3%]	24/1,674 [1.4%]	277/20,499 [1.3%]
**Health insurance:**	P<0.0001	P<0.0001	P<0.0001
**Yes**	33,169/207,706 [13.8%]	2,477/21,075 [10.5%]	30,692/18,6631 [14.1%]
**No**	4,900/8,304 [37.1%]	351/642 [35.3%]	4,549/7,662 [37.2%]
***ANY LIFETIME SUBSTANCE USE:***			
**Tobacco use:**	P<0.0001	P<0.0001	P<0.0001
**Yes**	21,942/79,384 [21.7%]	1,966/11,919 [14.1%]	19,976/67,465 [22.8%]
**No**	16,127/136,626 [10.6%]	862/9,798 [8.1%]	15,265/126,828 [10.7%]
**Alcohol consumption:**	P<0.0001	P<0.0001	P<0.0001
**Yes**	34,934/200,953 [14.8%]	2,691/21,035 [11.3%]	32,243/179,918 [15.2%]
**No**	3,135/15,057 [17.2%]	137/682 [16.7%]	2,998/14,375 [17.3%]
**Drug use:**	P<0.0001	P<0.0001	P<0.0001
**Yes**	18,217/112,271 [13.9%]	1,327/11,416 [10.4%]	16,890/100,855 [14.3%]
**No**	19,852/103,739 [16.0%]	1,501/10,301 [12.7%]	18,351/93,438 [16.4%]
***HEALTH:***			
**Self-rated health:**	P<0.0001	P<0.0001	P<0.0001
**Excellent**	2,363/28,416 [7.7%]	195/2,612 [6.9%]	2,168/25,804 [7.8%]
**Very good**	7,312/78,039 [8.6%]	601/8,198 [6.8%]	6,711/69,841 [8.8%]
**Good**	13,258/71,434 [15.6%]	1,008/7,218 [12.3%]	12,250/64,216 [16.0%]
**Fair**	11,271/31,696 [26.2%]	785/3,048 [20.5%]	10,486/28,648 [26.8%]
**Poor**	3,865/6,425 [37.6%]	239/641 [27.1%]	3,626/5,784 [38.5%]

*Abbreviations*: GED = General Education Development. ^a^ P values are based on Chi-square tests for independence. For employment status, the employed category comprises those labeled employed for wages or self-employed, and the unemployed category comprises those labeled as out of work, unable to work, retired, student, or homemaker. Percentages were rounded to one decimal place.

**Table 3** displays three multivariable logistic regression models for demographic, socioeconomic, substance use, and health characteristics as predictors of HI in the overall, veteran, and non-veteran populations. In fully adjusted models, male sex (Overall: adjusted odds ratio [AOR] = 1.39, 95% CI: 1.36,1.42; Veteran: AOR = 1.24, 95% CI: 1.11,1.40; Non-veteran: AOR = 1.40, 95% CI: 1.37,1.43), lifetime experience with cigarette smoking (Overall: AOR = 1.56, 95% CI: 1.54, 1.59; Veteran: AOR = 1.31, 95% CI: 1.19,1.44; Non-veteran: AOR = 1.58, 95% CI: 1.55,1.61), and worse self-rated health were associated with greater odds whereas increasing income level, and availability of health insurance (Overall: AOR = 0.69, 95% CI: 0.66,0.72; Veteran: AOR = 0.69, 95% CI: 0.59,0.82; Non-veteran: AOR = 0.69, 95% CI: 0.66,0.72) were associated with lower odds of HI, irrespective of veteran status. Regardless of veteran status, participants ≥70 years were less likely, and those 30–59 years were more likely, to experience HI compared to those 18–29 years of age. In addition, non-veterans 60–69 years were less likely to report HI than those 18–29 years of age. Among non-veterans only, the odds of HI were lower among those who reported their race as ‘Black’, ‘Asian’, ‘Multiple/Unknown’ versus ‘White’, slightly lower among those reporting any lifetime drug use, but higher among those reporting unemployment or any lifetime alcohol consumption. Regardless of veteran status, Hispanic/Latino ethnicity was unrelated to odds of HI, and participants who were never married, separated, divorced, or widowed, were more likely to experience HI compared to those who were married or living with a partner.

**Table 3:** Multivariable logistic regression models for participant characteristics as predictors of self-perceived housing instability over the past 6 months by veteran status–*All of Us* Research Program (n = 254,079) ^a^.

	Total (n = 254,079)	Veteran status	
		Veteran (n = 24,545)	Non-Veteran (n = 229,534)
	P AOR (95% CI)	P AOR (95% CI)	P AOR (95% CI)
***DEMOGRAPHIC:***			
**Sex:**			
**Female**	Ref.	Ref.	Ref.
**Male**	P<0.0001 1.39 (1.36, 1.42)	P = 0.0003 1.24 (1.11, 1.40)	P<0.0001 1.40 (1.37, 1.43)
**Age (years):**			
**18–29**	Ref.	Ref.	Ref.
**30–39**	P<0.0001 1.19 (1.12, 1.27)	P = 0.016 1.72 (1.11, 2.64)	P<0.0001 1.17 (1.13, 1.22)
**40–49**	P<0.0001 1.27 (1.19, 1.34)	P = 0.001 2.05 (1.33, 3.16)	P<0.0001 1.24 (1.17, 1.32)
**50–59**	P = 0.02 1.06 (1.00, 1.12)	P = 0.034 1.59 (1.04, 2.46)	P = 0.09 1.05 (0.99, 1.11)
**60–69**	P<0.0001 0.67 (0.64, 0.72)	P = 0.94 0.99 (0.64, 1.52)	P<0.0001 0.66 (0.62, 0.70)
**70–79**	P<0.0001 0.83 (0.78, 0.88)	P<0.0001 0.33 (0.22, 0.53)	P<0.0001 0.32 (0.30, 0.34)
**80+**	P<0.0001 0.17 (0.15, 0.19)	P<0.0001 0.19 (0.12, 0.31)	P<0.0001 0.19 (0.17, 0.21)
**Race:**			
**White**	Ref.	Ref.	Ref.
**Black**	P<0.0001 0.91 (0.88, 0.93)	P = 0.25 1.07 (0.95, 1.19)	P<0.0001 0.90 (0.88, 0.94)
**Asian**	P<0.0001 0.79 (0.73, 0.86)	P = 0.96 0.99 (0.64, 1.52)	P<0.0001 0.78 (0.72, 0.86)
**Other**	P = 0.93 0.99 (0.85, 1.16)	P = 0.11 1.68 (0.88, 3.21)	P = 0.59 0.96 (0.82, 1.13)
**Multiple / Unknown**	P<0.0001 0.87 (0.82, 0.93)	P = 0.35 1.11 (0.88, 1.41)	P<0.0001 0.87 (0.82, 0.92)
**Ethnicity:**			
**Hispanic or Latino**	Ref.	Ref.	Ref.
**Not Hispanic or Latino**	P = 0.16 1.04 (0.98, 1.10)	P = 0.64 1.06 (0.84, 1.34)	P = 0.19 1.04 (0.98, 1.10)
**Marital status:**			
**Never married**	P<0.0001 1.32 (1.27, 1.38)	P<0.0001 1.46 (1.27, 1.68)	P<0.0001 1.31 (1.26, 1.36)
**Married / Living with partner**	Ref.	Ref.	Ref.
**Separated / Divorced**	P<0.0001 1.69 (1.63, 1.77)	P<0.0001 1.79 (1.59, 2.01)	P<0.0001 1.68 (1.62, 1.77)
**Widowed**	P<0.0001 1.39 (1.31, 1.47)	P = 0.0006 1.44 (1.17, 1.79)	P<0.0001 1.36 (1.28, 1.44)
***SOCIOECONOMIC:***			
**Education:**			
**< High School**	Ref.	Ref.	Ref.
**High School / GED**	P = 0.19 1.03 (0.99, 1.07)	P = 0.90 1.02 (0.79, 1.32)	P = 0.27 1.02 (0.98, 1.06)
**Some College**	P<0.0001 1.15 (1.10, 1.19)	P = 0.76 1.04 (0.81, 1.34)	P<0.0001 1.15 (1.10, 1.19)
**College Graduate**	P = 0.69 0.99 (0.93, 1.05)	P = 0.83 0.97 (0.73, 1.27)	P = 0.51 0.99 (0.93, 1.05)
**Advanced degree**	P = 0.03 0.94 (0.88, 0.99)	P = 0.67 1.06 (0.79, 1.42)	P = 0.004 0.91 (0.86, 0.97)
**Employment status:**			
**Employed**	Ref.	Ref.	Ref.
**Unemployed**	P = 0.026 1.03 (1.01, 1.05)	P = 0.16 0.92 (0.82, 1.03)	P = 0.005 1.04 (1.00, 1.8)
**Income (U.S. dollars):**			
**< 10,000**	Ref.	Ref.	Ref.
**10,000–25,000**	P<0.0001 0.68 (0.66, 0.71)	P<0.0001 0.59 (0.53, 0.67)	P<0.0001 0.68 (0.66, 0.71)
**25,000–35,000**	P<0.0001 0.49 (0.48, 0.52)	P<0.0001 0.33 (0.28, 0.40)	P<0.0001 0.51 (0.49, 0.53)
**35,000–50,000**	P<0.0001 0.36 (0.35, 0.37)	P<0.0001 0.26 (0.22, 0.31)	P<0.0001 0.37 (0.35, 0.39)
**50,000–75,000**	P<0.0001 0.22 (0.21, 0.24)	P<0.0001 0.14 (0.12, 0.17)	P<0.0001 0.23 (0.22, 0.25)
**75,000–100,000**	P<0.0001 0.14 (0.13, 0.15)	P<0.0001 0.07 (0.05, 0.09)	P<0.0001 0.15 (0.14, 0.16)
**100,000–150,000**	P<0.0001 0.09 (0.08, 0.09)	P<0.0001 0.06 (0.05, 0.08)	P<0.0001 0.09 (0.08, 0.10)
**150,000–200,000**	P<0.0001 0.06 (0.05, 0.07)	P<0.0001 0.03 (0.02, 0.05)	P<0.0001 0.06 (0.06, 0.07)
**> 200,000**	P<0.0001 0.04 (0.04, 0.05)	P<0.0001 0.04 (0.03, 0.06)	P<0.0001 0.04 (0.04, 0.05)
**Health insurance:**			
**Yes**	P<0.0001 0.69 (0.66, 0.72)	P<0.0001 0.69 (0.59, 0.82)	P<0.0001 0.69 (0.66, 0.72)
**No**	Ref.	Ref.	Ref.
***ANY LIFETIME SUBSTANCE USE:***			
**Tobacco use:**			
**Yes**	P<0.0001 1.56 (1.54, 1.59)	P<0.0001 1.31 (1.19, 1.44)	P<0.0001 1.58 (1.55, 1.61)
**No**	Ref.	Ref.	Ref.
**Alcohol consumption:**			
**Yes**	P<0.0001 1.29 (1.25, 1.35)	P = 0.24 1.14 (0.92, 1.41)	P<0.0001 1.28 (1.23, 1.33)
**No**	Ref.	Ref.	Ref.
**Drug use:**			
**Yes**	P<0.0001 0.94 (0.92, 0.96)	P = 0.60 0.99 (0.88, 1.08)	P<0.0001 0.94 (0.92, 0.96)
**No**	Ref.	Ref.	Ref.
***HEALTH:***			
**Self-rated health:**			
**Excellent**	Ref.	Ref.	Ref.
**Very good**	P<0.0001 1.17 (1.13, 1.22)	P = 0.61 1.05 (0.88, 1.25)	P<0.0001 1.19 (1.13, 1.27)
**Good**	P<0.0001 1.62 (1.55, 1.68)	P = 0.0004 1.39 (1.17, 1.66)	P<0.0001 1.64 (1.55, 1.75)
**Fair**	P<0.0001 2.24 (2.12, 2.38)	P<0.0001 1.95 (1.64, 2.33)	P<0.0001 2.27 (2.14, 2.41)
**Poor**	P<0.0001 3.22 (3.09, 3.35)	P<0.0001 2.22 (2.14, 2.31)	P<0.0001 3.35 (3.17, 3.55)

*Abbreviations*: AOR = adjusted odds ratio; CI = Confidence interval; GED = General Education Development; ^a^ P values are based on Wald tests within multivariable logistic regression models for the overall, veteran, and non-veteran populations. For employment status, the employed category comprises those labeled employed for wages or self-employed, and the unemployed category comprises those labeled as out of work, unable to work, retired, student, or homemaker.

**Table 4** presents logistic regression models for veteran status as a predictor of HI, before and after sequentially controlling for demographic, socioeconomic, substance use, and health characteristics. The unadjusted model suggested that being a veteran was protective against HI (OR = 0.71, 95% CI: 0.69, 0.74). However, this relationship was attenuated but remained statistically significant after controlling for demographic characteristics alone (AOR = 0.94, 95% CI: 0.90, 0.98), and became statistically non-significant in the final model that controlled for all participant characteristics simultaneously (AOR = 0.97, 95% CI: 0.93, 1.01).

**Table 4:** Logistic regression models for veteran status as a predictor of self-perceived housing instability over the past 6 months–*All of Us* Research Program (n = 254,079) ^a^.

	OR (95% CI)
**Model 0: Unadjusted**	0.71 (0.69, 0.74)
**Model 1: Demographic**	0.94 (0.90, 0.98)
**Model 2: Demographic + Socioeconomic**	0.99 (0.95, 1.03)
**Model 3: Demographic + Socioeconomic + Substance use**	0.96 (0.92, 0.99)
**Model 4: Demographic + Socioeconomic + Substance use + Health**	0.97 (0.93, 1.01)

*Abbreviations*: CI = Confidence interval; OR = adjusted odds ratio; ^a^ Odds ratios and 95% confidence intervals are based on unadjusted (0) and adjusted (1, 2, 3, 4) logistic regression models for veteran status as a predictor of housing instability, with multivariable models sequentially adjusted for demographic, socioeconomic, substance use, and health characteristics.

## Discussion

HI is prevalent among both veteran and non-veteran populations in the U.S. [46–48]. Our analysis of cross-sectional data on more than 200,000 veteran and non-veteran participants from *AoU* found no significant differences in rates of HI over the past 6 months between veterans and non-veterans after controlling for various individual-level characteristics. Failure to reject the null hypothesis might suggest no inherent differences between veteran and non-veteran populations in terms of the burden of HI. Whereas veterans represent nearly 10% of homeless people in the U.S. [6], it may be important to consider that veterans may have unique vulnerabilities that predispose them to homelessness and HI, including those stemming from a history of combat exposure [3] or military sexual trauma [49], although these vulnerabilities may be counteracted by unique protective factors, such as access to health care benefits and other supportive services through the VA [3,47]. In general, we found that individuals between the ages of 30 and 59 years were more likely, and those 70 years and older were less likely, to experience HI compared to the youngest group aged between 18 and 29 years, with the age group between 60 and 69 being protected from HI among non-veterans only. Higher income and health insurance reduced the odds of HI, while male sex, unmarried status, lifetime cigarette smoking, and poorer self-rated health increased the odds. While Hispanic/Latino ethnicity was unrelated to HI, racial disparities that favor minoritized groups of non-veterans with regard to self-reported housing stability contradicts the preponderance of individuals who identify as Black among individuals experiencing homelessness [4,50]. For instance, analyses of Health and Retirement Study data estimated the lifetime prevalence of homelessness among Baby Boomers to be 6.2%, with rates of homelessness being higher among non-Hispanic blacks (16.8%) and Hispanics of any race (8.1%) than for non-Hispanic whites (4.8%) [51]. Our findings suggest distinct racial disparities between those studies that reported experiences of homeless individuals and our study participants who perceived HI. Another unexpected finding is that any lifetime drug use was weakly but inversely associated with HI among non-veterans, which is inconsistent with literature focused on homelessness [50,52–56]. Consistent with literature focused on homelessness, this study found that unemployment [46,53,57] and any lifetime alcohol consumption [23,53,58] were associated with increased risk for HI among non-veterans. These contradictory findings merit further investigation into distinct risk/protective factors associated with HI versus homelessness by veteran status. It is important to note that substance use disorders (SUDs) are a known risk factor for homelessness, but we broadly examined any use of alcohol and drugs in a participant’s lifetime, including even a one-time substance use experience. As such, our findings regarding substance use may not be comparable to those of studies that assessed more problematic substance use or SUDs.

Examination of HI correlates in this study was restricted by data availability and granularity within *AoU*, unlike previous studies that evaluated correlates of homelessness among U.S. veterans. For instance, a qualitative study highlighted childhood adversity, trauma/substance abuse during military service, post-military abuse, mental health, substance abuse, medical problems, unemployment, lack of social support, isolation, independence, and barriers to care, as characteristics that may initiate, promote, or reinforce homelessness among women veterans [46]. Similarly, a systematic review of 31 studies published between 1987 and 2014 on homelessness among U.S. veterans found that SUDs, mental illness, low income, social isolation, adverse childhood experiences, and past incarceration were the strongest risk factors, and that veterans, especially those who served since the advent of the all-volunteer force, were at greater risk for homelessness than other adults [48].

Correlates of HI have not been adequately examined in the general population of U.S. adults and have not been compared according to veteran status. Study findings were consistent with some, but not all, of the existing studies that evaluated correlates of HI among U.S. veterans. One study aimed to develop predictive models of HI and homelessness based on EHR data from 5.8 million veterans who responded to the VA’s Homelessness Screening Clinical Reminder, indicating that random forests models were more sensitive in predicting HI and homelessness than logistic regression, but less specific [59]. Another study involving 4,633,069 U.S. veterans found that sociodemographic and health service use variables were positively related to recent HI, with drug and opioid use disorders identified as key predictors of recent HI, thereby informing preventive interventions targeting new episodes of HI [60]. A study using VA data revealed that military sexual trauma, VA benefits inaccessibility, and single or divorced marital status were significant risk factors for HI among women veterans, aligning with a theoretical model emphasizing the importance of traumatic events and isolation, and suggesting new supportive interventions can mitigate their impact [61]. Using data on 38,633 post-9/11 U.S. veterans, a study found that one-third reported food and/or housing instability (FHI), with greater frequency among women and post-service [28]. Furthermore, FHI was associated with adverse childhood experiences, being enlisted, homelessness, depression, low social support, living with seriously ill/disabled persons, and living in dangerous neighborhoods [28]. Posttraumatic stress disorder, cholesterol level, hypertension, and illegal/street drug use were significant correlates of FHI among men, whereas morbid obesity and diabetes were significant correlates of FHI among women [28].

This study is among only a few that have used such a large sample to provide a contemporaneous picture of HI rather than homelessness among a racially and ethnically diverse group of veterans and non-veterans in the U.S., while extending previous research on this topic. Nevertheless, study findings should be interpreted taking several limitations into consideration. First, use of secondary data from the *AoU* restricted the scope of risk and protective characteristics evaluated in relation to HI and veteran status, and our ability to collect detailed information on alcohol consumption and drug use, or to re-define overlapping categories for annual household income. Second, the cross-sectional design precluded our ability to draw conclusions pertaining to the temporal relationship between the selected characteristics and HI or between veteran status and HI. Attempting to reduce the issue of reverse causation, we evaluated lifetime rather than current alcohol consumption and drug use in relation to recent experiences with HI. Given the dynamic nature of substance use and the fact that ever use of substances may not necessarily indicate SUDs, future studies should examine associations between diagnosed SUDs and HI among *AoU* participants with available EHR data. Third, volunteer bias is likely a concern since healthy individuals are more likely to enroll in the *AoU* and we did not use a nationally representative sample. VA facilities were among the HPOs that recruited *AoU* participants, but we cannot ascertain the proportion of VA-enrolled veterans in the study sample. Fourth, selection bias is likely because of eligibility criteria for participating in the *AoU* which restricted the population to individuals ≥ 18 years of age, with the population further restricted based on small cell sizes and availability of data on key variables, including veteran status, HI, and risk/protective characteristics. Non-response to surveys or items within surveys may have specifically resulted in selection bias. This issue is particularly relevant to the item used to define HI and its correlates. As shown in **S1 Table**, the proportion of veterans was slightly higher among *AoU* participants who were included in (9.7%) *vs*. excluded from (8.6%) complete case analyses. Similarly, the proportion experiencing HI was slightly lower among *AoU* participants who were included in (14.9%) *vs*. excluded from (18.9%) complete case analyses. Fifth, recall issues may have contributed to non-differential misclassification of variables of interest, potentially leading to underestimation of measures of association. Unlike nationally representative surveys which could potentially provide more accurate estimates of HI rates while examining trends in HI rate over time, the *AoU* is useful for examining associations of HI with risk/protective characteristics as well as health outcomes assessed using survey, physical measurement, bio-sample, and EHR data. A common limitation of these surveys as well as the *AoU* is that subjects who are currently homeless are likely missed and/or under-represented at the sampling stage. Importantly, HI and not homelessness was assessed, and as such, it is unknown how many of the subjects who experienced HI became homeless at a later stage. Sixth, residual confounding is likely since the use of secondary data from the *AoU* restricted our ability to control for additional covariates when examining the relationship between veteran status and HI. Seventh, the large sample size may have yielded statistically significant results that are not clinically meaningful based on their effect size. Eighth, the non-probability sampling strategy within the *AoU* precludes us from generalizing our findings to all non-institutionalized U.S. adults. Finally, *AoU* participants were enrolled during a period that spans the COVID-19 pandemic with variations in policymaking over time such as eviction moratoriums potentially affecting the estimated HI rate [62].

## Conclusions

There was no difference in rates of HI between veterans and non-veterans, suggesting HI is a relatively common phenomenon irrespective of veteran status. Furthermore, the burden of HI was shown to be multifactorial in nature. Certain demographic, socioeconomic, and health characteristics were associated with HI, with some of these modifiable and non-modifiable characteristics (*e*.*g*. race, employment, alcohol consumption, drug use) being more salient to non-veterans versus veterans, thereby informing the planning, implementation, and evaluation of evidence-based interventions aimed at addressing HI among veteran and non-veteran populations. Future research using *AoU* and VA data should examine the distinct correlates of HI and homelessness as well as the association between self-reported HI and future risk of homelessness among veteran and non-veteran populations. Studies comparing veterans at risk for homelessness to those already experiencing homelessness will likely inform VA programs such as the Supportive Services for Veterans and their Families (SSVF) and the Grant and Per Diem (GPD) programs. Given the cross-sectional nature of this study from a specific period of time, future research should incorporate time series analyses to examine external drivers influencing the proportion of veterans and non-veterans experiencing homelessness on an annual basis.

## Supporting information

S1 Table Comparison between *All of Us* Research Program participants included and excluded from the analysis on key characteristics. (DOCX)
