# Supplementary material for: Correlates of six-month housing instability among U.S. adults by veteran status: Exploratory study using data from the All of Us Program
Source: PLoS One. 2024 Nov 22;19(11):e0314339. doi: 10.1371/journal.pone.0314339 (PMC11584138; doi:10.1371/journal.pone.0314339)
Supplement: S1 Table — (DOCX) [file pone.0314339.s001.docx]

**SUPPORTING INFORMATION:**

**S1 Table.** Comparison between *All of Us* Research Program participants included and excluded from the analysis on key characteristics

|  | **Total** | **Included** | **Excluded** |
| --- | --- | --- | --- |
| *Veteran status:* |  | P<0.0001 | |
| Veteran | 36880 | 24545 | 12335 |
| Non-veteran | 361269 | 229534 | 131735 |
| *Housing instability:* |  | P<0.0001 | |
| Yes | 65298 | 38069 | 27229 |
| No | 332851 | 216010 | 116841 |
| *Sex:* |  | P=0.05 | |
| Female | 244035 | 156859 | 87176 |
| Male | 150534 | 97220 | 53314 |
| *Age (years):* |  |  |  |
|  |  | P<0.0001 | |
| 18-29 | 27609 | 16438 | 11171 |
| 30-39 | 60708 | 41001 | 19707 |
| 40-49 | 58790 | 38832 | 19958 |
| 50-59 | 66375 | 41499 | 24876 |
| 60-69 | 82853 | 52516 | 30337 |
| 70-79 | 69597 | 46417 | 23180 |
| 80+ | 28819 | 17376 | 11443 |
| *Race:* |  | P=0.87 | |
| White | 166027 | 165986 | 41 |
| Black | 41647 | 41637 | 10 |
| Asian | 9008 | 9007 | 1 |
| Other | 1681 | 1681 | 0 |
| Multiple / Unknown | 35778 | 35768 | 10 |
| *Ethnicity:* |  | P=0.72 | |
| Hispanic or Latino | 36792 | 36784 | 8 |
| Not Hispanic or Latino | 217349 | 217295 | 54 |
| *Marital status:* |  | P<0.0001 | |
| Never married | 99835 | 62196 | 37639 |
| Married / Living with partner | 200119 | 136707 | 63412 |
| Separated / Divorced | 68492 | 42927 | 25565 |
| Widowed | 21026 | 12249 | 8777 |
| *Education:* |  | P<0.0001 | |
| < High School | 34742 | 15670 | 19072 |
| High School / GED | 74245 | 39959 | 34286 |
| Some College | 101877 | 66249 | 35628 |
| College Graduate | 91849 | 65716 | 26133 |
| Advanced degree | 88037 | 66485 | 21552 |
| *Health insurance:* |  | P<0.0001 | |
| Yes | 363783 | 240875 | 122908 |
| No | 25654 | 13204 | 12450 |
| *Tobacco use:* |  | P=0.01 | |
| Yes | 150068 | 101326 | 48742 |
| No | 224956 | 152753 | 72203 |
| *Self-rated health:* |  | P<0.0001 | |
| Excellent | 44769 | 30779 | 13990 |
| Very Good | 119677 | 85351 | 34326 |
| Good | 130436 | 84692 | 45744 |
| Fair | 70783 | 42967 | 27816 |
| Poor | 17216 | 10290 | 6926 |
