# Supplementary material for: Correlates of six-month housing instability among U.S. adults by veteran status: Exploratory study using data from the All of Us Program
Source: PLoS One. 2024 Nov 22;19(11):e0314339. doi: 10.1371/journal.pone.0314339 (PMC11584138; doi:10.1371/journal.pone.0314339)
Supplement: Attachment — Submitted filename: PONE-D-24-25667 REVIEW.docx [file pone.0314339.s002.docx]

**PONE-D-24-25667 REVIEW**

Thank you for the opportunity to review this excellent research.

One minor revision recommended is that the Introduction briefly describe the difference between housing insecurity (HI) and homelessness for readers who may be unfamiliar with HI. As the authors state: “HI has been identified as a key predictor for homelessness.” A simple contrast could say: Someone who is housing insecure may be unable to pay rent or utilities, may live in substandard housing or overcrowded conditions, or need to move repeatedly. Homelessness, i.e., having no place to live, sleeping on the streets or in shelters, is the ultimate expression of housing insecurity.

Second minor revision is to tighten line spacing in the tables. Also consider eliminating all vertical borders while maintaining only the horizonal borders that separate key variables. These improvements will streamline tables and make them more legible.
