# Supplementary material for: Correlates of six-month housing instability among U.S. adults by veteran status: Exploratory study using data from the All of Us Program
Source: PLoS One. 2024 Nov 22;19(11):e0314339. doi: 10.1371/journal.pone.0314339 (PMC11584138; doi:10.1371/journal.pone.0314339)
Supplement: Attachment — Submitted filename: AOU_MAN_1_HI_PREDICTORS_DRAFT10_Response to Reviewers.docx [file pone.0314339.s003.docx]

**Response to Reviewers’ Comments**

**08/19/2024**

**PONE-D-24-25667**

**Correlates of six-month housing instability among U.S. adults by veteran status: Data from the All of Us Research Program**

**PLOS ONE

Dear Dr. Tsai,

Thank you for submitting your manuscript to PLOS ONE. After careful consideration, we feel that it has merit but does not fully meet PLOS ONE’s publication criteria as it currently stands. Therefore, we invite you to submit a revised version of the manuscript that addresses the points raised during the review process.

Please submit your revised manuscript by Sep 22 2024 11:59PM. If you will need more time than this to complete your revisions, please reply to this message or contact the journal office at** [**plosone@plos.org**](mailto:plosone@plos.org)**. When you're ready to submit your revision, log on to** [**https://www.editorialmanager.com/pone/**](https://www.editorialmanager.com/pone/) **and select the 'Submissions Needing Revision' folder to locate your manuscript file.

Please include the following items when submitting your revised manuscript:**

- **A rebuttal letter that responds to each point raised by the academic editor and reviewer(s). You should upload this letter as a separate file labeled 'Response to Reviewers'.**
- **A marked-up copy of your manuscript that highlights changes made to the original version. You should upload this as a separate file labeled 'Revised Manuscript with Track Changes'.**
- **An unmarked version of your revised paper without tracked changes. You should upload this as a separate file labeled 'Manuscript'.**

**If you would like to make changes to your financial disclosure, please include your updated statement in your cover letter. Guidelines for resubmitting your figure files are available below the reviewer comments at the end of this letter.

If applicable, we recommend that you deposit your laboratory protocols in protocols.io to enhance the reproducibility of your results. Protocols.io assigns your protocol its own identifier (DOI) so that it can be cited independently in the future. For instructions see:** [**https://journals.plos.org/plosone/s/submission-guidelines#loc-laboratory-protocols**](https://gcc02.safelinks.protection.outlook.com/?url=https%3A%2F%2Fc05y1x9s.r.us-east-2.awstrack.me%2FL0%2Fhttps%3A%252F%252Fjournals.plos.org%252Fplosone%252Fs%252Fsubmission-guidelines%2523loc-laboratory-protocols%2F1%2F010f0191332572ff-ba11ba70-6e5f-40a7-860a-9e434bade637-000000%2FKHqUc_PE2kU4FpXw7qifYJS1z-w%3D171&data=05%7C02%7C%7C3ae95ec07fb946ba97f108dcb8198b10%7Ce95f1b23abaf45ee821db7ab251ab3bf%7C0%7C0%7C638587668638317552%7CUnknown%7CTWFpbGZsb3d8eyJWIjoiMC4wLjAwMDAiLCJQIjoiV2luMzIiLCJBTiI6Ik1haWwiLCJXVCI6Mn0%3D%7C0%7C%7C%7C&sdata=40kbz2GrYEfEv0IoHJ7h6F7rSdCHYWChTLitqq6bmBU%3D&reserved=0)**. Additionally, PLOS ONE offers an option for publishing peer-reviewed Lab Protocol articles, which describe protocols hosted on protocols.io. Read more information on sharing protocols at** [**https://plos.org/protocols?utm_medium=editorial-email&utm_source=authorletters&utm_campaign=protocols**](https://gcc02.safelinks.protection.outlook.com/?url=https%3A%2F%2Fc05y1x9s.r.us-east-2.awstrack.me%2FL0%2Fhttps%3A%252F%252Fplos.org%252Fprotocols%253Futm_medium%3Deditorial-email%2526utm_source%3Dauthorletters%2526utm_campaign%3Dprotocols%2F1%2F010f0191332572ff-ba11ba70-6e5f-40a7-860a-9e434bade637-000000%2Fzo4mOTIvoYwaHN_C7WtfuWX9vNo%3D171&data=05%7C02%7C%7C3ae95ec07fb946ba97f108dcb8198b10%7Ce95f1b23abaf45ee821db7ab251ab3bf%7C0%7C0%7C638587668638327970%7CUnknown%7CTWFpbGZsb3d8eyJWIjoiMC4wLjAwMDAiLCJQIjoiV2luMzIiLCJBTiI6Ik1haWwiLCJXVCI6Mn0%3D%7C0%7C%7C%7C&sdata=CVqHNqESgP%2BiuFUWVODZVBfgVahHg1UgaxEhutiTlYo%3D&reserved=0)**.

We look forward to receiving your revised manuscript.**

**Kind regards,**

**Lakshminarayana Chekuri, MD, PhD
Academic Editor
PLOS ONE**

**Journal requirements:**

**1. When submitting your revision, we need you to address these additional requirements.**

**Please ensure that your manuscript meets PLOS ONE's style requirements, including those for file naming. The PLOS ONE style templates can be found at** 
[**https://journals.plos.org/plosone/s/file?id=wjVg/PLOSOne_formatting_sample_main_body.pdf**](https://journals.plos.org/plosone/s/file?id=wjVg/PLOSOne_formatting_sample_main_body.pdf) **and** 
[**https://journals.plos.org/plosone/s/file?id=ba62/PLOSOne_formatting_sample_title_authors_affiliations.pdf**](https://journals.plos.org/plosone/s/file?id=ba62/PLOSOne_formatting_sample_title_authors_affiliations.pdf)**.**

**2. We note that you have indicated that there are restrictions to data sharing for this study. PLOS only allows data to be available upon request if there are legal or ethical restrictions on sharing data publicly. For more information on unacceptable data access restrictions, please see** [**http://journals.plos.org/plosone/s/data-availability#loc-unacceptable-data-access-restrictions**](http://journals.plos.org/plosone/s/data-availability#loc-unacceptable-data-access-restrictions)**.**

**Before we proceed with your manuscript, please address the following prompts:**

**a) If there are ethical or legal restrictions on sharing a de-identified data set, please explain them in detail (e.g., data contain potentially identifying or sensitive patient information, data are owned by a third-party organization, etc.) and who has imposed them (e.g., a Research Ethics Committee or Institutional Review Board, etc.). Please also provide contact information for a data access committee, ethics committee, or other institutional body to which data requests may be sent.**

**b) If there are no restrictions, please upload the minimal anonymized data set necessary to replicate your study findings to a stable, public repository and provide us with the relevant URLs, DOIs, or accession numbers. For a list of recommended repositories, please see**
[**https://journals.plos.org/plosone/s/recommended-repositories**](https://journals.plos.org/plosone/s/recommended-repositories)**. You also have the option of uploading the data as Supporting Information files, but we would recommend depositing data directly to a data repository if possible.**

**We will update your Data Availability statement on your behalf to reflect the information you provide.**

**3. Please include your full ethics statement in the ‘Methods’ section of your manuscript file. In your statement, please include the full name of the IRB or ethics committee who approved or waived your study, as well as whether or not you obtained informed written or verbal consent. If consent was waived for your study, please include this information in your statement as well.**

**Additional Editor Comments:

Thank you for your scholarly contribution to the topic area of Homelessness. I'd also like to thank the authors for choosing PLOS ONE to publish your findings from this study. My comments and comments from three other experts in this topic area are provided below. Please review these comments and I suggest address them and resubmit your manuscript. Your timely response would help this study be published and will make it accessible to interested readers across the world. I look forward to reviewing your revised manuscript. I wish you good luck with your future endeavors.

Editor comments:

Comment #1: I'd recommend adding "Homelessness" as one of the keywords.**

**RESPONSE: We acknowledge the editor’s comment. Accordingly, we have added homelessness as one of the keywords listed after the Abstract.**

**Comment #2: The authors' description that this study used a "comprehensive dataset" appears to be somewhat misleading (page 29). For instance, by the authors' own acknowledgement (page 5) "we restricted the study to participants ≥ 18 years with no missing data on key variables." This suggests that only a partial dataset was used for this analysis. On page 5, the authors state that "9,939 participants" were excluded for various reasons. This could have affected the findings in this study. Such limitations need to be acknowledged in the manuscript.**

**RESPONSE: We acknowledge the editor’s comment. Accordingly, we revised a sentence within the last paragraph of the Discussion section by deleting the phrase “comprehensive dataset”, as follows:**

***“This study is among few that have used such a large sample to provide a contemporaneous picture of HI rather than homelessness among a racially and ethnically diverse group of veterans and non-veterans in the U.S., while extending previous research on this topic.”***

**Furthermore, we added details to the limitations sub-section of the Discussion section in order to address selection bias that may have resulted from the eligibility criteria within the All Of Us Research Program and our study, as follows:**

***“Fourth, selection bias is likely because of eligibility criteria for participating in the AoU Research Program which restricted the population to individuals ≥ 18 years of age, with the population further restricted based on small cell sizes and availability of data on key variables, including veteran status, HI, and risk/protective characteristics. Non-response to surveys or items within surveys may have specifically resulted in selection bias.”***

**Comment #3: On Page 11, Table 1, socio-economic section the authors present data on income as discrete categories. However, the groups seem to be overlapping. For instance, respondents earning US$ 25000 have a likelihood of being grouped twice. The same pattern is noted in tables 2 and 3 as well. Please address this.

RESPONSE: We acknowledge the editor’s comment. To clarify, we are performing secondary analyses of existing data from the *All of Us* Research Program. The documentation provided online by the *All of Us* Research Program for the “Household income” item from the “Basics” survey through the Data Browser (**[**View Survey Questions and Answers | All of Us Public Data Browser (researchallofus.org)**](https://databrowser.researchallofus.org/survey/the-basics)**) suggests that these are response categories were as follows: ‘Annual income: less than 10k’, ‘Annual income: 10k 25k’, ‘Annual income: 25k 35k’, ‘Annual income: 35k 50k’, ‘Annual income: 50k 75k’, ‘Annual income: 75k 100k’, ‘Annual income: 100k 150k’, ‘Annual income: 100k 150k’, ‘Annual income: 150k 200k’, ‘Annual income: more 200k’, ‘Skip’, ‘Prefer Not to Answer’. After removing missing data on this variable, we were able to translate these labels as follows: < 10,000, 10,000-25,000, 25,000-35,000, 35,000-50,000, 50,000-75,000, 75,000-100,000, 100,000-150,000, and > 200,000. Since we are researchers using secondary data from the All Of Us Research Program, we added a limitation within the Discussion section that generally pertains to secondary data use, while highlighting the issue of annual household income categories, as follows:**

***“First, use of secondary data from the AoU Research Program restricted the scope of risk and protective characteristics evaluated in relation to HI and veteran status, and our ability to collect detailed information on alcohol consumption and drug use, or to re-define overlapping categories for annual household income.”***

**Comment #4: On Page 7, under substance use and health characteristics you describe one of the questions as “In your entire life, have you had at least 1 drink of any kind of alcohol, not counting small tastes or sips?” Please explain the relevance of "at least 1 drink of any kind of alcohol" consumption and its influence on HI. Did the authors hypothesize that all respondents who consumed "at least 1 drink of any kind of alcohol" in their "entire life" to have an alcohol use disorder and there by carried a higher risk for HI? Likewise, I cannot understand the relevance of "LIFETIME" "EVER USAGE" of drugs and its relation to HI. Please acknowledge these as limitations for this study.**

**RESPONSE: We acknowledge the editor’s comment. Accordingly, we revised the limitations sub-section within the Discussion section to clarify our choices of study variables to define alcohol consumption and drug use, as well as opportunities for future analyses within the All of Us Research Program:**

***“Attempting to reduce the issue of reverse causation, we evaluated lifetime rather than current alcohol consumption and drug use in relation to recent experiences with HI. Given the dynamic nature of substance use and the fact that ever use of substances may not necessarily indicate a SUDs, future studies should examine associations between diagnosed SUDs and HI among AoU Research Program participants with available EHR data.”***

**Also, for the question "In your LIFETIME, which of the following substances have you ever used?”, please detail what kind of substances the study screened for.**

**RESPONSE: We acknowledge the editor’s comment. Accordingly, we added details regarding drug use, *i.e.,* the types of substances that were screened for within the “Lifestyle” survey of the All of Us Research Program as follows:**

***“Using the “Lifestyle” survey component we defined any lifetime history of substance use as dichotomous (Yes or No) variables for cigarette smoking (“Have you smoked at least 100 cigarettes in your entire life?”), alcohol consumption (“In your entire life, have you had at least 1 drink of any kind of alcohol, not counting small tastes or sips?”) and drug use (“In your lifetime, which of the following substances have you ever used?”), with drug types categorized as marijuana, hallucinogens, cocaine, methamphetamines, prescription stimulants, sedatives, prescription opioids, street opioids, inhalants, and other drugs. Notably, any lifetime use of a substance, in this context, included even a one-time use”.***

**Finally, as some of the reviewers rightly pointed out, the study has significant limitations, and such limitations need to be acknowledged in the manuscript.**

**RESPONSE: We acknowledge the editor’s comments. Accordingly, we made every effort to address and/or acknowledge these study limitations, as indicated by the reviewers.**

**Please see an acknowledgment of these limitations pertaining to substance use within the Discussion section:**

***“It is important to note that substance use disorders (SUDs) are a known risk factor for homelessness, but we broadly examined any use of alcohol and drugs in a participant’s lifetime, including even a one-time substance use experience. A such, our findings regarding substance use may not be comparable to those of studies that assessed more problematic substance use or SUDs.”***

***“Second, the cross-sectional design precludes our ability to draw conclusions pertaining to the temporal relationship between the selected characteristics and HI or between veteran status and HI. Attempting to reduce the issue of reverse causation, we evaluated lifetime rather than current alcohol consumption and drug use in relation to recent experiences with HI. Given the dynamic nature of substance use and the fact that ever use of substances may not necessarily indicate a SUDs, future studies should examine associations between diagnosed SUDs and HI among AoU Research Program participants with available EHR data.”***

**Reviewers' comments:

Reviewer's Responses to Questions**

**Comments to the Author

1. Is the manuscript technically sound, and do the data support the conclusions?

The manuscript must describe a technically sound piece of scientific research with data that supports the conclusions. Experiments must have been conducted rigorously, with appropriate controls, replication, and sample sizes. The conclusions must be drawn appropriately based on the data presented.**

**Reviewer #1: Partly**

**Reviewer #2: Yes**

**Reviewer #3: Yes**

**2. Has the statistical analysis been performed appropriately and rigorously?**

**Reviewer #1: No**

**Reviewer #2: Yes**

**Reviewer #3: Yes**

**3. Have the authors made all data underlying the findings in their manuscript fully available?

The** [**PLOS Data policy**](https://gcc02.safelinks.protection.outlook.com/?url=http%3A%2F%2Fc05y1x9s.r.us-east-2.awstrack.me%2FL0%2Fhttp%3A%252F%252Fwww.plosone.org%252Fstatic%252Fpolicies.action%2523sharing%2F1%2F010f0191332572ff-ba11ba70-6e5f-40a7-860a-9e434bade637-000000%2FJ7sVQJYvh5-Ejg7X607481WIqTQ%3D171&data=05%7C02%7C%7C3ae95ec07fb946ba97f108dcb8198b10%7Ce95f1b23abaf45ee821db7ab251ab3bf%7C0%7C0%7C638587668638335494%7CUnknown%7CTWFpbGZsb3d8eyJWIjoiMC4wLjAwMDAiLCJQIjoiV2luMzIiLCJBTiI6Ik1haWwiLCJXVCI6Mn0%3D%7C0%7C%7C%7C&sdata=Ox3Pe1oyN9OXIswLYvqm8nB1PAUUtFgcWdfh9zwueN0%3D&reserved=0) **requires authors to make all data underlying the findings described in their manuscript fully available without restriction, with rare exception (please refer to the Data Availability Statement in the manuscript PDF file). The data should be provided as part of the manuscript or its supporting information, or deposited to a public repository. For example, in addition to summary statistics, the data points behind means, medians and variance measures should be available. If there are restrictions on publicly sharing data—e.g. participant privacy or use of data from a third party—those must be specified.**

**Reviewer #1: Yes**

**Reviewer #2: Yes**

**Reviewer #3: Yes**

**4. Is the manuscript presented in an intelligible fashion and written in standard English?

PLOS ONE does not copyedit accepted manuscripts, so the language in submitted articles must be clear, correct, and unambiguous. Any typographical or grammatical errors should be corrected at revision, so please note any specific errors here.**

**Reviewer #1: Yes**

**Reviewer #2: Yes**

**Reviewer #3: Yes**

**5. Review Comments to the Author

Please use the space provided to explain your answers to the questions above. You may also include additional comments for the author, including concerns about dual publication, research ethics, or publication ethics. (Please upload your review as an attachment if it exceeds 20,000 characters)**

**Reviewer #1:**

**Comment #1: This manuscript focuses on housing insecurity (HI) and veteran status based on analysis of a large survey dataset. While this promises some interesting and important findings, I found the paper conceptually muddled and unfocused. I’m recommending some major revisions.**

**RESPONSE: We acknowledge the reviewer’s comments. Accordingly, we made every effort to focus the paper based on the stated objectives and performed major revisions, as requested.**

**Comment #2: First, from the manuscript’s lead sentence, the authors link previous research on HI (which is sparse) with research on homelessness (which is much more plentiful), especially insofar as it involves veterans. Although they are careful to differentiate the two overlapping phenomena, they at least implicitly promise insights into homelessness with this analysis with sentences such as “updated studies may be needed to maintain a current understanding of homelessness among veterans” (62-63) that it simply never delivers. Nor does it need to as HI is a topic well worth additional study by itself. Instead of linking HI with homelessness, I urge the authors to consider linking it to homeless prevention, if in no other way that both phenomena involve a population that appears to be at imminent risk of becoming homeless, but most manage to maintain their housing (however precarious) and avoid homelessness.**

**RESPONSE: We acknowledge the reviewer’s comments. We made several changes within the Introduction and Discussion sections that clarify the concepts of homelessness and HI, as well as evidence pertaining to these concepts. To emphasize the link between HI and homelessness prevention, we revised the sentence the reviewer is referring to as follows:**

***“Accordingly, up-to-date studies may be needed to maintain a current understanding of HI for the purpose of homelessness prevention through services provided to veteran and non-veteran populations.”***

**Comment #3: Looked at in a veteran context, the subgroup considered as HI in this paper (persons worried about losing their housing) would be more germane to a constituency served by Supportive Services for Veterans and their Families (SSVF) than by an interim housing program such as Grant and Per Diem (GPD).**

**RESPONSE: We acknowledge the reviewer’s comment. Accordingly, we have added a sentence within the Conclusion section to address relevance to VA programs, as shown below:**

***“Studies comparing veterans at risk for homelessness to those already experiencing homelessness will likely inform VA programs such as the ‘Supportive Services for Veterans and their Families’ and the ‘Grant and Per Diem’ regarding their constituencies.”***

**Comment #4: Second, the paper never explains why veteran and non-veteran populations could be expected to differ in the prevalence of HI experiences. Beyond the Fargo et al. study cited in the manuscript, there is little evidence to support a higher prevalence of homelessness among non-veterans, and there is also a paradox that I’m sure the authors are aware where veterans should be at lower risk for homelessness due to better economic, employment and educational outcomes, and a more robust social safety net due to the VA. Additionally, given the relatively advanced mean age of the survey respondents, for many of the respondents there has been a time lag spanning multiple decades between their military service and recent HI, which would attenuate associations between the two experiences.**

**RESPONSE: We acknowledge the reviewer’s comment. Accordingly, we added a sentence within the Introduction section that explains why veterans may differ in terms of HI experiences than their non-veteran counterparts, as follows:**

***“U.S. veterans constitute a special population that may be at increased risk for HI and homelessness because of detrimental experiences before, during, and after military service, although these risks may paradoxically be mitigated by demographic and socioeconomic characteristics that favor veterans and the availability of social services through the U.S. Department of Veterans Affairs (VA) [9].”***

**Comment #5: Beyond this, there is little rationale given for examining relationships between HI and the other characteristics that become covariates for the analyses. What results is a study that is basically exploratory, and comes up with an aggregation of characteristics that are associated with HI rather than a clear narrative or framework that these characteristics could fit into. This leads to the first two paragraphs of the discussion section, which I find difficult to find any clear “take-home” (sic) message. Even the study’s main finding, that there was no association found between veteran status and HI experience, is undercut by the authors’ ex post facto equivocations. So why do the study at all?**

**RESPONSE: We acknowledge the reviewer’s comment. To clarify, this study is exploratory in nature, and was conducted due the scarcity of evidence pertaining to HI. The main purpose of this study is to provide a picture for the epidemiology of HI by examining demographic, socioeconomic, substance use, and health characteristics among veteran and non-veteran populations, as stated within the Introduction section. We believe the association between veteran status and HI is not significant and that is notable (i.e., null results are informative). As such, we revised several sentences within the Introduction section, as follows:**

***“Recent studies focused on HI and homelessness among veteran and non-veteran populations have identified a wide range of socio-demographic (e.g. sex, age, race/ethnicity, education, income, health insurance, foster care, entitlements, veteran status), behavioral (e.g. smoking status, alcohol consumption, substance use, sexual practices, sensation-seeking, aggression) and clinical (e.g. body mass index, chronic conditions, physical/mental health) correlates for these outcomes[4-6, 21]. Unlike homelessness which is the ultimate expression of HI, evidence pertaining to HI among veterans has been limited, with several studies focused on evaluating HI screening and intervention activities, and fewer of these studies focused on identifying risk/protective factors for HI[6, 11, 22-36]. In this exploratory study, we used data from the All of Us Research Program (AoU) – a unique database funded by the U.S. National Institute of Health – to examine the epidemiology of HI among U.S. veteran and non-veteran adult participants. We aimed to address the following research questions in our analyses: [1] which demographic, socioeconomic, substance use, and health characteristics previously linked to homelessness are associated with HI among veteran and non-veterans? and [2] Is veteran status associated with HI, net of other factors? Study findings are expected to contribute to the current understanding of HI epidemiology by veteran status.”***

**Furthermore, we revised and combined the first two paragraphs of the Discussion section for the purpose of clarity when it comes to the relationship between veteran status and HI, as shown below:**

***“HI remains prevalent among both veteran and non-veteran populations in the U.S.[44-46]. Our analysis of cross-sectional data on more than 200,000 veteran and non-veteran participants from AoU found no significant differences in rates of HI over the past 6 months between veterans and non-veterans after controlling for various individual-level characteristics. Whereas veterans represent nearly 10% of homeless people in the U.S. [5], it may be important to consider that veterans may have unique vulnerabilities that predispose them to homelessness and HI, including those stemming from a history of combat exposure[9] or military sexual trauma[47], although these vulnerabilities may be counteracted by unique protective factors, such as access to health care and case management supports through the VA[9, 45]. In general, we found that individuals between the ages of 30 and 59 years were more likely, and those 70 years and older were less likely, to experience HI compared to the youngest group aged between 18 and 29 years, with the age group between 60 and 69 being protected from HI among non-veterans only. Higher income and health insurance reduced the odds of HI, while male sex, unmarried status, lifetime cigarette smoking, and poorer self-rated health increased the odds. While Hispanic/Latino ethnicity was unrelated to HI, racial disparities that favor minoritized groups with regard to self-reported housing stability contradicts the preponderance of individuals who identify as Black among individuals who experience homelessness[3, 48], suggesting distinct racial disparities between those who experience homelessness and those at-risk for experiencing homelessness. Another unexpected finding is that any lifetime drug was weakly but inversely associated with HI among non-veterans[48-53]. Conversely, this study found that unemployment[44, 50, 54] and any lifetime alcohol consumption[21, 50, 55] were associated with increased risk for HI among non-veterans. These contradictory findings merit further investigation into distinct risk/protective factors for HI and homelessness by veteran status. It is important to note that substance use disorders (SUDs) are a known risk factor for homelessness, but we broadly examined any use of alcohol and drugs in a participant’s lifetime, including even a one-time substance use experience. A such, our findings regarding substance use may not be comparable to those of studies that assessed more problematic substance use or SUDs.”***

**Comment #6: Additionally, many of the significant associations between covariates and HI experience that the authors highlight are statistically significant due to the large n of the study group, but are arguably non-substantial based on the magnitude of the odds ratio. The authors need to address this.

RESPONSE: We acknowledge the reviewer’s comment. Accordingly, we added a sentence within the limitations sub-section of the Discussion section to address the issue of large sample size, as shown below:**

***“Seventh, the large sample size may have yielding statistically significant results that are not clinically meaningful based on their effect size.”***

**Comment #7: Finally, the authors gloss over several substantial findings in their discussion of the results that would call into question whether HI experience involved similar dynamics as experiences of homelessness. The most opaque of these is race, in which the authors compare all races to Asian, which makes the racial differences harder to compare and obscures the lack of apparent differences between Black and White races in the odds of experiencing HI. This would be very different from homelessness, where being black almost universally increases the risk for homelessness across many studies. Similarly, the non-significant or extremely modest associations between alcohol and substance use and HI experience runs counter to the relationships generally found between these covariates and homelessness.**

**RESPONSE: We acknowledge the reviewer’s comment. Accordingly, we revised the Results section and analyses within Table 3, whereby the referent category is no longer the ‘Asian’ group but the ‘White’ group since the majority of the study sample falls within that group. As such, we are now comparing all other racial groups (including the ‘Black’ group) to the ‘White’ group, as shown below:**

***“Among non-veterans only, the odds of HI were lower among those who reported their race as ‘Black’, ‘Asian’, ‘Multiple/Unknown’ versus ‘White’, slightly lower among those reporting any lifetime drug use, but higher among those reporting unemployment or any lifetime alcohol consumption.”***

**We also revised the Discussion section as follows:**

***“While Hispanic/Latino ethnicity was unrelated to HI, racial disparities that favor minoritized groups with regard to self-reported housing stability contradicts the preponderance of individuals who identify as Black among individuals who experience homelessness[3, 48], suggesting distinct racial disparities between those who experience homelessness and those at-risk for experiencing homelessness. Another unexpected finding is that any lifetime drug was weakly but inversely associated with HI among non-veterans[48-53]. Conversely, this study found that unemployment[44, 50, 54] and any lifetime alcohol consumption[21, 50, 55] were associated with increased risk for HI among non-veterans. These contradictory findings merit further investigation into distinct risk/protective factors for HI and homelessness by veteran status. It is important to note that substance use disorders (SUDs) are a known risk factor for homelessness, but we broadly examined any use of alcohol and drugs in a participant’s lifetime, including even a one-time substance use experience. A such, our findings regarding substance use may not be comparable to those of studies that assessed more problematic substance use or SUDs.”***

**Comment #8: Put all of this together, and I really can’t figure out what I should take away from this study.**

**RESPONSE: We acknowledge the reviewer’s comment. In the revised version of the manuscript, we have made every effort to provide a clearer rationale for conducting this study, while addressing editorial and reviewer comments.**

**Reviewer #2: Thank you for the opportunity to review this important research providing a timely, comprehensive account of HI among veterans and non-veterans in the U.S.

Comment #1: One minor revision recommended is that the Introduction briefly describe the difference between housing insecurity (HI) and homelessness for readers who may be unfamiliar with HI. As the authors state: “HI has been identified as a key predictor for homelessness.” A simple contrast could say: Someone who is housing insecure may be unable to pay rent or utilities, may live in substandard housing or overcrowded conditions, or need to move repeatedly. Homelessness, i.e., having no place to live, sleeping on the streets or in shelters, is the ultimate expression of housing insecurity.**

**RESPONSE: We acknowledge the reviewer’s comment. Accordingly, we provided a brief definition of homelessness and housing instability in the first paragraph of the Introduction, as shown below:**

***“Housing instability (HI), commonly defined by researchers and practitioners as the state of living in housing but being at-risk of losing housing due to challenges such as difficulties paying rent, overcrowding, frequent relocation, and substantial proportion of income spent on housing [1, 2], is a pathway to homelessness defined as lack of stable, safe, and functioning housing.”***

**We also revised a sentence within the Introduction section as follows:**

***“Unlike homelessness which is the ultimate expression of HI, evidence pertaining to HI among veterans has been limited, with several studies focused on evaluating HI screening and intervention activities, and fewer of these studies focused on identifying risk/protective factors for HI[6, 11, 22-36].”***

**Comment #2: Second minor revision is to tighten line spacing in the tables. Also consider eliminating all vertical borders while maintaining only the horizonal borders that separate key variables. These improvements will streamline tables and make them more legible.**

**RESPONSE: We acknowledge the reviewer’s comment. Accordingly, we eliminated all vertical borders while maintaining only horizontal borders separating key variables and tightened line spacing within the tables.**

**Reviewer #3: This manuscript outlines a secondary data analysis of a large survey-based dataset to compare rates and correlates of housing instability in US Veterans and non-Veterans. Overall, I found this paper to be well organized and its topic to make a significant contribution to the broader literature. I have only minor comments as listed below:

INTRODUCTION**

*** Use of symbols in the opening paragraph is distracting and reduces readability. Please replace symbols (e.g., instead of ">," spell out "more than"; instead of "~," spell out "approximately")
* Line 66 seems to have a typo - "suggesting" should be "suggested"
* Please include conceptualizations/operationalizations of homelessness and housing instability in the Introduction to better orient the reader to the topic of study. These conceptualizations should also serve to highlight the unique contribution of this study to the broader literature given its focus on housing instability rather than homelessness.**

**RESPONSE: We acknowledge the reviewer’s comments. Accordingly, we spelled symbols such as “>” and "~," throughout the manuscript. We also corrected the error in line 66. Finally, we included a conceptualization and operationalization of homelessness and housing instability in the first paragraph of the manuscript, as shown below:**

***“Housing instability (HI), commonly defined by researchers and practitioners as the state of living in housing but being at-risk of losing housing due to challenges such as difficulties paying rent, overcrowding, frequent relocation, and substantial proportion of income spent on housing [1, 2], is a pathway to homelessness defined as lack of stable, safe, and functioning housing.”***

**METHOD**

*** Please see comment above regarding use of symbols
* Line 101 suggests participants with missing data were excluded from the sample; however, line 102-103 appears to contradict this by implying that further participants were excluded due to skipping an item about housing instability. Please clarify.
* In describing individual items, it is unclear why some words are provided in all caps.**

**RESPONSE: We acknowledge the reviewer’s comments. Accordingly, we spelled symbols such as “>” and "~," throughout the manuscript. To clarify, we excluded participants with missing data on any of the key characteristics, regardless of whether they skipped an item or they provided a non-informative response. We revised the text as follows:**

***“After excluding 9,939 participants because they had missing data on the question pertaining to HI, a total of 398,149 participants were considered study eligible.”***

**Finally, we removed the ‘all caps’ when describing specific items within the Methods section.**

**RESULTS**

*** What do authors make of the exceptionally high rates of unemployment? Unemployment rates appear particularly at odds with reported annual incomes
* Line 164 reads as if only 3.3% and 7.6% of participants reported any history of alcohol consumption; please reword to reflect that these proportions denied history of alcohol consumption (or instead include statistics for those reporting history of alcohol consumption)**

**RESPONSE: We acknowledge the reviewer’s comment. Accordingly, we added the following sentence within the Methods section that clarifies how employment status was defined:**

***“Whereas the employed category comprises those labeled employed for wages or self-employed, the unemployed category comprises those labeled as out of work, unable to work, retired, student, or homemaker.”***

**Table footnotes were updated accordingly.**

**Within the Results section, we rephrased as follows to clarify findings that pertain to alcohol consumption:**

***“ … or lacking any lifetime alcohol consumption (3.3% vs. 7.6%)”***

**DISCUSSION**

*** Given the limitations to sampling procedures, I would discourage authors from making claims about prevalence within the discussion (e.g., line 311).
* Can authors speak more to implications of the current study's focus on housing instability rather than homelessness? There is some comparison of results to those of studies focusing on homelessness; however, these are two distinct constructs and additional discussion about implications for housing instability specifically would be helpful.**

**RESPONSE: We acknowledge the reviewer’s comments. Accordingly, we revised the Discussion and conclusion sections as follows:**

**Discussion:**

***“Our analysis of cross-sectional data on more than 200,000 veteran and non-veteran participants from AoU found no significant differences in rates of HI over the past 6 months between veterans and non-veterans after controlling for various individual-level characteristics.”***

**Conclusion:**

***“There was no difference in rates of HI between veterans and non-veterans, suggesting HI is a common phenomenon irrespective of veteran status.”***

**Furthermore, we agree with the reviewer that homelessness and HI are two distinct constructs, and this issue was raised by another reviewer. In the revised version of the manuscript, we made several changes throughout the manuscript to incorporate more discussion of the literature on HI in addition to the existing literature on homelessness.**

**For instance, we added the following sentences within the Discussion section:**

***“A recent study involving 38,633 post-9/11 U.S. veterans found that one-third reported food and/or housing instability (FHI), with greater frequency among women and post-service [26]. Furthermore, FHI was associated with adverse childhood experiences, being enlisted, homelessness, depression, low social support, living with seriously ill/disabled persons, and living in dangerous neighborhoods[26]. Posttraumatic stress disorder, cholesterol level, hypertension, and illegal/street drug use were significant correlates of FHI among men, whereas morbid obesity and diabetes were significant correlates of FHI among women[26].”***

**We also revised the following sentence as follows within the Introduction section:**

***“Unlike homelessness which is the ultimate expression of HI, evidence pertaining to HI among veterans has been limited, with several studies focused on evaluating HI screening and intervention activities, and fewer of these studies focused on identifying risk/protective factors for HI[6, 11, 22-36]”.***

**Please note that we could not find any published article that specifically focuses on risk/protective factors for HI.**

**Accordingly, we revised a sentence to the Discussion section as follows:**

***“This study is among few that have used such a large sample to provide a contemporaneous picture of HI rather than homelessness among a racially and ethnically diverse group of veterans and non-veterans in the U.S., while extending previous research on this topic.”***

**6. PLOS authors have the option to publish the peer review history of their article (**[**what does this mean?**](https://gcc02.safelinks.protection.outlook.com/?url=https%3A%2F%2Fc05y1x9s.r.us-east-2.awstrack.me%2FL0%2Fhttps%3A%252F%252Fjournals.plos.org%252Fplosone%252Fs%252Feditorial-and-peer-review-process%2523loc-peer-review-history%2F1%2F010f0191332572ff-ba11ba70-6e5f-40a7-860a-9e434bade637-000000%2FzSpPBOjnsRPuuCo7K0cPHQ5craU%3D171&data=05%7C02%7C%7C3ae95ec07fb946ba97f108dcb8198b10%7Ce95f1b23abaf45ee821db7ab251ab3bf%7C0%7C0%7C638587668638341007%7CUnknown%7CTWFpbGZsb3d8eyJWIjoiMC4wLjAwMDAiLCJQIjoiV2luMzIiLCJBTiI6Ik1haWwiLCJXVCI6Mn0%3D%7C0%7C%7C%7C&sdata=KQQlvs0KFgpW6cCPc19v4VQdTBwz6%2FKlyDFZu9QWW04%3D&reserved=0)**). If published, this will include your full peer review and any attached files.


If you choose “no”, your identity will remain anonymous but your review may still be made public.


Do you want your identity to be public for this peer review? For information about this choice, including consent withdrawal, please see our** [**Privacy Policy**](https://gcc02.safelinks.protection.outlook.com/?url=https%3A%2F%2Fc05y1x9s.r.us-east-2.awstrack.me%2FL0%2Fhttps%3A%252F%252Fwww.plos.org%252Fprivacy-policy%2F1%2F010f0191332572ff-ba11ba70-6e5f-40a7-860a-9e434bade637-000000%2FonA73zzoYpEX5-hKJNHiAHftxQQ%3D171&data=05%7C02%7C%7C3ae95ec07fb946ba97f108dcb8198b10%7Ce95f1b23abaf45ee821db7ab251ab3bf%7C0%7C0%7C638587668638346975%7CUnknown%7CTWFpbGZsb3d8eyJWIjoiMC4wLjAwMDAiLCJQIjoiV2luMzIiLCJBTiI6Ik1haWwiLCJXVCI6Mn0%3D%7C0%7C%7C%7C&sdata=%2BFFnIW%2BL8afhJqSw3AVZ%2F2CyBL8cqxhV55pFv9Uk0J4%3D&reserved=0)**.**

**Reviewer #1: Yes: Steve Metraux**

**Reviewer #2: No**

**Reviewer #3: Yes: Emily Edwards**

**[NOTE: If reviewer comments were submitted as an attachment file, they will be attached to this email and accessible via the submission site. Please log into your account, locate the manuscript record, and check for the action link "View Attachments". If this link does not appear, there are no attachment files.]

While revising your submission, please upload your figure files to the Preflight Analysis and Conversion Engine (PACE) digital diagnostic tool,** [**https://pacev2.apexcovantage.com/**](https://gcc02.safelinks.protection.outlook.com/?url=https%3A%2F%2Fc05y1x9s.r.us-east-2.awstrack.me%2FL0%2Fhttps%3A%252F%252Fpacev2.apexcovantage.com%252F%2F1%2F010f0191332572ff-ba11ba70-6e5f-40a7-860a-9e434bade637-000000%2FaI4GyRkKhjO44YrEipe_0C-HhzM%3D171&data=05%7C02%7C%7C3ae95ec07fb946ba97f108dcb8198b10%7Ce95f1b23abaf45ee821db7ab251ab3bf%7C0%7C0%7C638587668638352781%7CUnknown%7CTWFpbGZsb3d8eyJWIjoiMC4wLjAwMDAiLCJQIjoiV2luMzIiLCJBTiI6Ik1haWwiLCJXVCI6Mn0%3D%7C0%7C%7C%7C&sdata=teCAgd6sE1W2uDZ7pdHBHV%2Fq4UPQkKQ3U1AUeJtpPcs%3D&reserved=0)**. PACE helps ensure that figures meet PLOS requirements. To use PACE, you must first register as a user. Registration is free. Then, login and navigate to the UPLOAD tab, where you will find detailed instructions on how to use the tool. If you encounter any issues or have any questions when using PACE, please email PLOS at** [**figures@plos.org**](mailto:figures@plos.org)**. Please note that Supporting Information files do not need this step.**
