# Supplementary material for: Correlates of six-month housing instability among U.S. adults by veteran status: Exploratory study using data from the All of Us Program
Source: PLoS One. 2024 Nov 22;19(11):e0314339. doi: 10.1371/journal.pone.0314339 (PMC11584138; doi:10.1371/journal.pone.0314339)
Supplement: Attachment — Submitted filename: PONE_R&R_R1_093024.docx [file pone.0314339.s004.docx]

**Response to Reviewer’s Comments:**

**09/30/2024**

**From:** [**em.pone.0.8e5af9.47097968@editorialmanager.com**](mailto:em.pone.0.8e5af9.47097968@editorialmanager.com) **<**[**em.pone.0.8e5af9.47097968@editorialmanager.com**](mailto:em.pone.0.8e5af9.47097968@editorialmanager.com)**>**

**On Behalf Of PLOS ONE
Sent: Friday, September 27, 2024 2:32 AM
To: Tsai, Jack <**[**Jack.Tsai@uth.tmc.edu**](mailto:Jack.Tsai@uth.tmc.edu)**>
Subject: PLOS ONE Decision: Revision required [PONE-D-24-25667R1] - [EMID:81f8ed56a6510214]**

**External: Increase caution when handling links and attachments.**

**CC:** [**lchekuri@kansascity.edu**](mailto:lchekuri@kansascity.edu)**,** [**lchekuri@freemanhealth.com**](mailto:lchekuri@freemanhealth.com)

**PONE-D-24-25667R1**

**Correlates of six-month housing instability among U.S. adults by veteran status: Data from the All of Us Research Program**

**PLOS ONE

Dear Dr. Tsai,

Thank you for submitting your manuscript to PLOS ONE. After careful consideration, we feel that it has merit but does not fully meet PLOS ONE’s publication criteria as it currently stands. Therefore, we invite you to submit a revised version of the manuscript that addresses the points raised during the review process.

Please submit your revised manuscript by Nov 11 2024 11:59PM. If you will need more time than this to complete your revisions, please reply to this message or contact the journal office at** [**plosone@plos.org**](mailto:plosone@plos.org)**. When you're ready to submit your revision, log on to** [**https://www.editorialmanager.com/pone/**](https://gcc02.safelinks.protection.outlook.com/?url=https%3A%2F%2Fwww.editorialmanager.com%2Fpone%2F&data=05%7C02%7C%7Cdd4764da64cc4e33791108dcdf04d1d1%7Ce95f1b23abaf45ee821db7ab251ab3bf%7C0%7C0%7C638630459011408741%7CUnknown%7CTWFpbGZsb3d8eyJWIjoiMC4wLjAwMDAiLCJQIjoiV2luMzIiLCJBTiI6Ik1haWwiLCJXVCI6Mn0%3D%7C0%7C%7C%7C&sdata=X%2BvFsq3WgJHwsOso41vImN5ejQwkGNACvLeFfCrvNTk%3D&reserved=0) **and select the 'Submissions Needing Revision' folder to locate your manuscript file.

Please include the following items when submitting your revised manuscript:**

- **A rebuttal letter that responds to each point raised by the academic editor and reviewer(s). You should upload this letter as a separate file labeled 'Response to Reviewers'.**
- **A marked-up copy of your manuscript that highlights changes made to the original version. You should upload this as a separate file labeled 'Revised Manuscript with Track Changes'.**
- **An unmarked version of your revised paper without tracked changes. You should upload this as a separate file labeled 'Manuscript'.**

**If you would like to make changes to your financial disclosure, please include your updated statement in your cover letter. Guidelines for resubmitting your figure files are available below the reviewer comments at the end of this letter.

If applicable, we recommend that you deposit your laboratory protocols in protocols.io to enhance the reproducibility of your results. Protocols.io assigns your protocol its own identifier (DOI) so that it can be cited independently in the future. For instructions see:** [**https://journals.plos.org/plosone/s/submission-guidelines#loc-laboratory-protocols**](https://gcc02.safelinks.protection.outlook.com/?url=https%3A%2F%2Fc05y1x9s.r.us-east-2.awstrack.me%2FL0%2Fhttps%3A%252F%252Fjournals.plos.org%252Fplosone%252Fs%252Fsubmission-guidelines%2523loc-laboratory-protocols%2F1%2F010f019232647c43-f0b13182-6b73-4324-ade4-df0e61340e0c-000000%2FYtKarehoMsvAruzLLrZgFs1xM_0%3D178&data=05%7C02%7C%7Cdd4764da64cc4e33791108dcdf04d1d1%7Ce95f1b23abaf45ee821db7ab251ab3bf%7C0%7C0%7C638630459011428311%7CUnknown%7CTWFpbGZsb3d8eyJWIjoiMC4wLjAwMDAiLCJQIjoiV2luMzIiLCJBTiI6Ik1haWwiLCJXVCI6Mn0%3D%7C0%7C%7C%7C&sdata=Ey2x5uoKr03HvCgsMnsMIlbhTEDWNDOOIPsV80U%2FEjw%3D&reserved=0)**. Additionally, PLOS ONE offers an option for publishing peer-reviewed Lab Protocol articles, which describe protocols hosted on protocols.io. Read more information on sharing protocols at** [**https://plos.org/protocols?utm_medium=editorial-email&utm_source=authorletters&utm_campaign=protocols**](https://gcc02.safelinks.protection.outlook.com/?url=https%3A%2F%2Fc05y1x9s.r.us-east-2.awstrack.me%2FL0%2Fhttps%3A%252F%252Fplos.org%252Fprotocols%253Futm_medium%3Deditorial-email%2526utm_source%3Dauthorletters%2526utm_campaign%3Dprotocols%2F1%2F010f019232647c43-f0b13182-6b73-4324-ade4-df0e61340e0c-000000%2FyBG7jjNxLDSeX9jw3awPAfW1yMQ%3D178&data=05%7C02%7C%7Cdd4764da64cc4e33791108dcdf04d1d1%7Ce95f1b23abaf45ee821db7ab251ab3bf%7C0%7C0%7C638630459011442291%7CUnknown%7CTWFpbGZsb3d8eyJWIjoiMC4wLjAwMDAiLCJQIjoiV2luMzIiLCJBTiI6Ik1haWwiLCJXVCI6Mn0%3D%7C0%7C%7C%7C&sdata=nZhyiH9KSrzJWX0NmoNABO8VMIaeyiAykUpdryWpARo%3D&reserved=0)**.

We look forward to receiving your revised manuscript.**

**Kind regards,**

**Lakshminarayana Chekuri, MD, PhD
Academic Editor
PLOS ONE**

**Journal Requirements:

Please review your reference list to ensure that it is complete and correct. If you have cited papers that have been retracted, please include the rationale for doing so in the manuscript text, or remove these references and replace them with relevant current references. Any changes to the reference list should be mentioned in the rebuttal letter that accompanies your revised manuscript. If you need to cite a retracted article, indicate the article’s retracted status in the References list and also include a citation and full reference for the retraction notice.**

**RESPONSE: In the process of revising this manuscript, we reviewed the reference list to ensure that it is complete and correct. Furthermore, we ensured that none of the references cited have been retracted. Finally, we would like to indicate that several references were either added or deleted from the reference list based on comments provided by the editor and reviewers.**

**Additional Editor Comments:

Thank you for your scholarly contribution to the topic area of Homelessness. I'd also like to thank the authors for choosing PLOS ONE to publish your findings from this study. I believe this manuscript is getting close to acceptance for a publication. My comments and comments from reviewer 1 are provided below. Please review these comments and I suggest address them and resubmit your manuscript. Your timely response would help this study be published and accessible to interested readers across the world. I look forward to reviewing your revised manuscript. I wish you good luck with your future endeavors.

Editor comments:

line 53 – Please consider adding "in the U.S." at the end of the sentence**

**RESPONSE: We agree with the editorial comment. Accordingly, we added “in the U.S.” at the end of the sentence on line 53, as follows:**

***“In 2023, it was estimated that more than 600,000 individuals were homeless on a given night in the U.S. [10].”***

**lines 89-90: You state "Unlike homelessness which is the ultimate expression of HI....", please provide a citation for your assertion "homelessness .... is the ultimate expression of HI".**

**RESPONSE: We acknowledge the editorial comment. Accordingly, we provided a citation for the assertion that “homelessness .... is the ultimate expression of HI” on line 89-90, and revised the sentence as follows:**

***“HI is an important social determinant of health affecting adults in the U.S. [4-7], and homelessness, defined as lack of stable, safe, and functioning housing, is a severe manifestation of HI [8, 9].”***

**The reference is shown below.**

[**https://www.ncbi.nlm.nih.gov/pmc/articles/PMC6445694/**](https://www.ncbi.nlm.nih.gov/pmc/articles/PMC6445694/)

***https://health.gov/healthypeople/priority-areas/social-determinants-health/literature-summaries/housing-instability#:~:text=Evictions%20that%20go%20through%20the,8%2C20*

Table 2: please consider adding a statement that states percentages were rounded to first decimal.**

**RESPONSE: We acknowledge the editorial comment. Accordingly, we added the following statement as a footnote to Table 2:**

***“Percentages were rounded to one decimal place”***

**I could not find "Figure 1" that is referenced across the manuscript. Please provide this for my review.

RESPONSE: We acknowledge the editorial comment. In the revised version of the manuscript, we made sure to attach Figure 1 to the submission for review.**

**In the "materials and methods" section (line 100): If available, please provide response rate and total number of surveys sent out.**

**RESPONSE: We acknowledge the editorial comment. We would like to clarify that, unlike nationally representative surveys, the All of Us Research Program enrolled subjects on a voluntary basis. Also, the All of Us surveys that we analyzed in this study were required rather than optional surveys. Therefore, response rates for these surveys was high. Please see below the revised sentence within the Methods section:**

***“Self-reported data were obtained on a subset of 397,191 participants who completed the three non-optional surveys, namely, “The Basics”, “Lifestyle”, and “Overall Health”, with a response rate of 99.8% among study-eligible participants enrolled in the AoU program.”.***

**On line 290: Please correct typo "A such" to "As such"**

**RESPONSE: We acknowledge the editorial comment. Accordingly, we revised “A such” to “As such” on line 290.**

**The abbreviation "VA" was used throughout the manuscript. I could not find the full expansion of this abbreviation in the manuscript. I'd recommend provide a full expansion of this abbreviation at the first instance.

RESPONSE: We acknowledge the editorial comment. Please note that we had provided a full expansion of “VA” abbreviation at the first instance of use, as follows:**

***“U.S. veterans constitute a special population that may be at increased risk for HI because of detrimental experiences before, during, and after military service, although these risks may be partly mitigated by demographic and socioeconomic characteristics that favor veterans and the availability of health and social services through the U.S. Department of Veterans Affairs (VA) [3].”***

**[Note: HTML markup is below. Please do not edit.]**

**Reviewers' comments:

Reviewer's Responses to Questions**

**Comments to the Author

1. If the authors have adequately addressed your comments raised in a previous round of review and you feel that this manuscript is now acceptable for publication, you may indicate that here to bypass the “Comments to the Author” section, enter your conflict of interest statement in the “Confidential to Editor” section, and submit your "Accept" recommendation.**

**Reviewer #1: (No Response)**

**Reviewer #2: All comments have been addressed**

**2. Is the manuscript technically sound, and do the data support the conclusions?

The manuscript must describe a technically sound piece of scientific research with data that supports the conclusions. Experiments must have been conducted rigorously, with appropriate controls, replication, and sample sizes. The conclusions must be drawn appropriately based on the data presented.**

**Reviewer #1: Partly**

**Reviewer #2: Yes**

**3. Has the statistical analysis been performed appropriately and rigorously?**

**Reviewer #1: Yes**

**Reviewer #2: Yes**

**4. Have the authors made all data underlying the findings in their manuscript fully available?

The** [**PLOS Data policy**](https://gcc02.safelinks.protection.outlook.com/?url=http%3A%2F%2Fc05y1x9s.r.us-east-2.awstrack.me%2FL0%2Fhttp%3A%252F%252Fwww.plosone.org%252Fstatic%252Fpolicies.action%2523sharing%2F1%2F010f019232647c43-f0b13182-6b73-4324-ade4-df0e61340e0c-000000%2FJe02vEfscYl5SzGVzmosi9G6Bls%3D178&data=05%7C02%7C%7Cdd4764da64cc4e33791108dcdf04d1d1%7Ce95f1b23abaf45ee821db7ab251ab3bf%7C0%7C0%7C638630459011455500%7CUnknown%7CTWFpbGZsb3d8eyJWIjoiMC4wLjAwMDAiLCJQIjoiV2luMzIiLCJBTiI6Ik1haWwiLCJXVCI6Mn0%3D%7C0%7C%7C%7C&sdata=NE5J%2BIRn6Q9w1weElSD2Aa%2Fj9r0auoof2lQPt8wNAYA%3D&reserved=0) **requires authors to make all data underlying the findings described in their manuscript fully available without restriction, with rare exception (please refer to the Data Availability Statement in the manuscript PDF file). The data should be provided as part of the manuscript or its supporting information, or deposited to a public repository. For example, in addition to summary statistics, the data points behind means, medians and variance measures should be available. If there are restrictions on publicly sharing data—e.g. participant privacy or use of data from a third party—those must be specified.**

**Reviewer #1: (No Response)**

**Reviewer #2: Yes**

**5. Is the manuscript presented in an intelligible fashion and written in standard English?

PLOS ONE does not copyedit accepted manuscripts, so the language in submitted articles must be clear, correct, and unambiguous. Any typographical or grammatical errors should be corrected at revision, so please note any specific errors here.**

**Reviewer #1: Yes**

**Reviewer #2: Yes**

**6. Review Comments to the Author

Please use the space provided to explain your answers to the questions above. You may also include additional comments for the author, including concerns about dual publication, research ethics, or publication ethics. (Please upload your review as an attachment if it exceeds 20,000 characters)**

**Reviewer #1: I appreciate the authors’ attentiveness to my concerns and comments regarding the original version of their manuscript. In responding to their revised text, I’ll group my comments by those pertaining to the introductory text and then to the text in the discussion/conclusion sections.

Regarding the introductory text, I still have problems with how the authors frame the study. When I commented, in my initial review, that the authors frame this study in the context of housing insecurity and homelessness prevention instead of in terms of actual homelessness (which this study really doesn’t address), I meant the authors do more than just rephrase a few passages and insert the term “housing insecurity” into a few places while keeping the initial references and keeping the structure of the introduction largely unchanged.

On reading the revised manuscript, the disconnect between HI, as the authors look at it and what the data contains, and homelessness is more stark. This is not a homelessness paper at all, as the large majority of those who express HI, such as is examined in this manuscript, will never go on to experience homelessness and the data for this study has no indicator for actual homelessness. Thus, for example, the numbers the authors cite about the incidence of veteran homelessness are largely irrelevant to the paper. The authors’ attempt to connect the two gets even more tenuous when their stated definition of HI, as presented at the start of the manuscript, conflicts with their working definition of homelessness as “the ultimate expression of homelessness” (see line 98-99). Finally, while there is not a large literature on HI, there is a literature on this that includes research both on housing instability (see for example AE Montgomery and colleagues’ work with the VA housing screener) and on homeless prevention that directly informs what the authors are looking at here and needs to be included to set the context for the study at hand.**

**RESPONSE: We thank the reviewer for the constructive feedback on the revised manuscript. Accordingly, we re-wrote the introduction, discussion, and concluding sections to address the reviewer’s comments. Specifically, the focus of thes sections is no longer on actual homelessness, but rather on housing instability and homelessness prevention.**

**We agree with the reviewer that there is a disconnect between housing instability and actual homelessness, since that majority of individuals who experience housing instability will never experience homelessness. Furthermore, we agree that the All Of Us Research Program data cannot be used to examine actual homelessness, and that the definition provided regarding housing instability conflicts with that of homelessness. As such, we revised the Introduction to focus more on housing instability and homelessness prevention, and to better provide context for this study. Please note that we have cited work related to housing instability in the Introduction section, including work by Montgomery and colleagues, and also presented the most relevant work focused on housing instability in the Discussion section. Please see track-changes within the Introduction and Discussion sections.**

**The sentence that the authors inserted (“U.S. veterans constitute…”) in response to what they designated as my “comment #4” would do well as the lead sentence in the manuscript rather than be buried in the middle of a long first paragraph.**

**RESPONSE: We acknowledge the reviewer’s comment. In the revised version of the manuscript, the sentence mentioned by the reviewer is the second sentence of the first paragraph of the Introduction.**

***“U.S. veterans constitute a special population that may be at increased risk for HI because of detrimental experiences before, during, and after military service, although these risks may be partly mitigated by demographic and socioeconomic characteristics that favor veterans and the availability of health and social services through the U.S. Department of Veterans Affairs (VA) [3].”***

**Moving to the comments pertaining to the material in the Discussion and Conclusion sections, the authors main finding is that they fail to find a significant difference in the risk for HI between veterans and nonveterans (and the unadjusted difference actually has significantly lower rates of HI for veterans). In explaining this, they speculate on a balancing out of risk factors and protective factors among veterans. But in doing this they fail to consider the simpler option, failing to reject the implicit null hypothesis that there is no evidence of any inherent veteran/non-veteran difference in risk for HI. This latter explanation is the more parsimonious one, and in justifying their alternative explanation on offsetting protective and risk factors, they use research on homelessness for support where there is no evidence to equate risk for HI with risk of homelessness, and also mention a study on risk factors for HI among veterans that, if such a study in fact exists, they would need to cite that study rather than the review article they currently cite to back up their assertion that “unique vulnerabilities” may predispose veterans to HI.**

**RESPONSE: We agree with the reviewer that emphasis should be placed on failing to reject the null hypothesis of a difference in the prevalence of housing instability between veterans and non-veterans, after controlling for confounders, rather than emphasize the balancing out of risk factors and protective factors among veterans which can also lead to this finding. In the revised version of the Discussion section, we wrote:**

***“Failure to reject the null hypothesis might suggest no inherent differences between veteran and non-veteran populations in terms of the burden of HI.”***

**Furthermore, we provided further literature that is focused on housing instability rather than homelessness, including the study on risk factors for HI among veterans, which is more relevant than the review article on homelessness, which was moved to another paragraph within the Discussion section. Please see track-changes within the paragraph:**

***“Correlates of HI have not been adequately examined in the general population of U.S. adults and have not been compared according to veteran status. Study findings were consistent with some, but not all, of the existing studies that evaluated correlates of HI among U.S. veterans. One study aimed to develop predictive models of HI and homelessness based on electronic medical record data from 5.8 million veterans who responded to the VA's Homelessness Screening Clinical Reminder, indicating that random forests models were more sensitive in predicting HI and homelessness than logistic regression, but less specific [59]. Another study involving 4,633,069 U.S. veterans found that sociodemographic and health service use variables were positively related to recent HI, with drug and opioid use disorders identified as key predictors of recent HI, thereby informing preventive interventions targeting new episodes of HI [60]. A study using VA data revealed that military sexual trauma, VA benefits inaccessibility, and single or divorced marital status were significant risk factors for HI among women veterans, aligning with a theoretical model emphasizing the importance of traumatic events and isolation, and suggesting new supportive interventions can mitigate their impact [61]. Using data on 38,633 post-9/11 U.S. veterans, a study found that one-third reported food and/or housing instability (FHI), with greater frequency among women and post-service [28]. Furthermore, FHI was associated with adverse childhood experiences, being enlisted, homelessness, depression, low social support, living with seriously ill/disabled persons, and living in dangerous neighborhoods [28]. Posttraumatic stress disorder, cholesterol level, hypertension, and illegal/street drug use were significant correlates of FHI among men, whereas morbid obesity and diabetes were significant correlates of FHI among women [28].”***

**Beyond that, the conclusions they make are, as they now acknowledge, exploratory and focus on risk for HI more generally (i.e., among both veteran and nonveteran populations). I appreciate their attentiveness to my comments about findings related to race, substance use, and significance and effect size, however, the main message I get from the discussion is that there are an overwhelming number of factors that indicate some degree of significant risk for homelessness without a clear framework for organizing the risk or much of how to translate these risks to more applied settings.**

**RESPONSE: We acknowledge the reviewer’s comment. Accordingly, the concluding paragraph was revised to emphasize modifiable and non-modifiable correlates of housing instability for the purpose of homelessness prevention among veteran and non-veteran populations. We emphasized the multifactorial nature of housing instability, and provided practical implications to study findings.**

***“Furthermore, the burden of HI was shown to be multifactorial in nature. Certain demographic, socioeconomic, substance use, and health characteristics were associated with HI, with some of these modifiable and non-modifiable characteristics (e.g. race, employment, alcohol consumption, drug use) being more salient to non-veterans versus veterans, thereby informing the planning, implementation, and evaluation of evidence-based interventions aimed at addressing HI among veteran and non-veteran populations.”***

**In summary, to respond to my continued concerns would still require major revisions along the lines of the issues that I initially raised in my first review.**

**RESPONSE: We acknowledge the reviewer’s comments. Accordingly, major revisions were made to the manuscript, taking into consideration the comments provided by the reviewer at first review.**

**Reviewer #2: Thank you for addressing this reviewer's comments. The revised manuscript is technically sound, the data support the conclusions, and the writing is articulate. The statistical analysis been performed appropriately and rigorously. Underlying findings are made available.**

**RESPONSE: We thank the reviewer for the positive feedback on the revised manuscript.**

**7. PLOS authors have the option to publish the peer review history of their article (**[**what does this mean?**](https://gcc02.safelinks.protection.outlook.com/?url=https%3A%2F%2Fc05y1x9s.r.us-east-2.awstrack.me%2FL0%2Fhttps%3A%252F%252Fjournals.plos.org%252Fplosone%252Fs%252Feditorial-and-peer-review-process%2523loc-peer-review-history%2F1%2F010f019232647c43-f0b13182-6b73-4324-ade4-df0e61340e0c-000000%2FCfLDoHtaViw5RidTvDyHXa_Az_g%3D178&data=05%7C02%7C%7Cdd4764da64cc4e33791108dcdf04d1d1%7Ce95f1b23abaf45ee821db7ab251ab3bf%7C0%7C0%7C638630459011468873%7CUnknown%7CTWFpbGZsb3d8eyJWIjoiMC4wLjAwMDAiLCJQIjoiV2luMzIiLCJBTiI6Ik1haWwiLCJXVCI6Mn0%3D%7C0%7C%7C%7C&sdata=AX%2FNCOqbve1jSmfTlH0nNHwQqcwtfebQxJVo87q9A4g%3D&reserved=0)**). If published, this will include your full peer review and any attached files.


If you choose “no”, your identity will remain anonymous but your review may still be made public.


Do you want your identity to be public for this peer review? For information about this choice, including consent withdrawal, please see our** [**Privacy Policy**](https://gcc02.safelinks.protection.outlook.com/?url=https%3A%2F%2Fc05y1x9s.r.us-east-2.awstrack.me%2FL0%2Fhttps%3A%252F%252Fwww.plos.org%252Fprivacy-policy%2F1%2F010f019232647c43-f0b13182-6b73-4324-ade4-df0e61340e0c-000000%2Fazc6XPpkTOSlIKC07xHmJPmU_G4%3D178&data=05%7C02%7C%7Cdd4764da64cc4e33791108dcdf04d1d1%7Ce95f1b23abaf45ee821db7ab251ab3bf%7C0%7C0%7C638630459011482348%7CUnknown%7CTWFpbGZsb3d8eyJWIjoiMC4wLjAwMDAiLCJQIjoiV2luMzIiLCJBTiI6Ik1haWwiLCJXVCI6Mn0%3D%7C0%7C%7C%7C&sdata=Ei0q%2Fgchb9HVhArStRR9fZRuCUc9TwAuLBoKXYeyU1k%3D&reserved=0)**.**

**Reviewer #1: Yes: Stephen Metraux**

**Reviewer #2: No**

**[NOTE: If reviewer comments were submitted as an attachment file, they will be attached to this email and accessible via the submission site. Please log into your account, locate the manuscript record, and check for the action link "View Attachments". If this link does not appear, there are no attachment files.]

While revising your submission, please upload your figure files to the Preflight Analysis and Conversion Engine (PACE) digital diagnostic tool,** [**https://pacev2.apexcovantage.com/**](https://gcc02.safelinks.protection.outlook.com/?url=https%3A%2F%2Fc05y1x9s.r.us-east-2.awstrack.me%2FL0%2Fhttps%3A%252F%252Fpacev2.apexcovantage.com%252F%2F1%2F010f019232647c43-f0b13182-6b73-4324-ade4-df0e61340e0c-000000%2FQTsgMatYYUep6fIZNhgDKw5VOD8%3D178&data=05%7C02%7C%7Cdd4764da64cc4e33791108dcdf04d1d1%7Ce95f1b23abaf45ee821db7ab251ab3bf%7C0%7C0%7C638630459011497474%7CUnknown%7CTWFpbGZsb3d8eyJWIjoiMC4wLjAwMDAiLCJQIjoiV2luMzIiLCJBTiI6Ik1haWwiLCJXVCI6Mn0%3D%7C0%7C%7C%7C&sdata=ohbfjN%2F0A%2FiOzrDL4TmBmMkPrse%2Fw%2FcNcOIwce4hQ4k%3D&reserved=0)**. PACE helps ensure that figures meet PLOS requirements. To use PACE, you must first register as a user. Registration is free. Then, login and navigate to the UPLOAD tab, where you will find detailed instructions on how to use the tool. If you encounter any issues or have any questions when using PACE, please email PLOS at** [**figures@plos.org**](mailto:figures@plos.org)**. Please note that Supporting Information files do not need this step.**
