# Supplementary material for: Correlates of six-month housing instability among U.S. adults by veteran status: Exploratory study using data from the All of Us Program
Source: PLoS One. 2024 Nov 22;19(11):e0314339. doi: 10.1371/journal.pone.0314339 (PMC11584138; doi:10.1371/journal.pone.0314339)
Supplement: Attachment — Submitted filename: Editor comments 11-03-24 PONE-D-24-25667.docx [file pone.0314339.s005.docx]

Thank you for your scholarly contribution to the topic area of Homelessness. I'd also like to thank the authors for choosing PLOS ONE to publish your findings from this study. I am tentatively accepting this manuscript for publication pending some minor recommended changes as described below. Please review these comments and I suggest addressing them and resubmit your manuscript. Your timely response would help this study be published and will make it accessible to interested readers across the world. I wish you good luck with your future endeavors.

**Editor – suggested changes:**

Line 1: Consider adding "**Exploratory study of**" Data from ..... in the title.

Line 25, Consider adding "**(U.S)**" at the end of this sentence. I see that you used "U.S" to reflect United States from here onwards.

Line 80: consider changing "chronic conditions" to "chronic **health** conditions"

Line 102, expand "EHR" and abbreviate in parenthesis, since this is the first time "EHR" is being used in the manuscript.

Line 156, consider rephrasing "Using the “Lifestyle” survey component we defined any …..”

to "Using the “Lifestyle” survey component we ~~defined~~ **assessed** any…..”

Line 285: Consider rephrasing the following statement: "Our findings suggest distinct racial disparities between those who experience homelessness and those who report being at risk of experiencing homelessness based on HI." The contents of your current statement “……and **those who report being at risk of experiencing homelessness** based on HI” appear a bit misleading since your study did not find a relationship between HI and homelessness.

I'd recommend as follows: "**Our findings suggest distinct racial disparities between those studies that reported experiences of homeless individuals and our study participants who perceived HI.**”

Line 366: consider changing "yielding" to "**yielded**"

Line 375: Since this is a concluding statement, I'd recommend consider deleting "substance use" from this sentence. I am recommending this because your study's assessment of substance use has serious limitations, as you rightly acknowledged.
