# Supplementary material for: Correlates of six-month housing instability among U.S. adults by veteran status: Exploratory study using data from the All of Us Program
Source: PLoS One. 2024 Nov 22;19(11):e0314339. doi: 10.1371/journal.pone.0314339 (PMC11584138; doi:10.1371/journal.pone.0314339)
Supplement: Peer review history

# Peer review: Decision Letter 0

**DOI:** 10.1371/journal.pone.0314339.r001

**Authors:** Lakshminarayana Chekuri

8 Aug 2024

PONE-D-24-25667Correlates of six-month housing instability among U.S. adults by veteran status: Data from the All of Us Research ProgramPLOS ONE

Dear Dr. Tsai,

Thank you for submitting your manuscript to PLOS ONE. After careful consideration, we feel that it has merit but does not fully meet PLOS ONE’s publication criteria as it currently stands. Therefore, we invite you to submit a revised version of the manuscript that addresses the points raised during the review process.

Please submit your revised manuscript by Sep 22 2024 11:59PM. If you will need more time than this to complete your revisions, please reply to this message or contact the journal office at plosone@plos.org. When you're ready to submit your revision, log on to https://www.editorialmanager.com/pone/ and select the 'Submissions Needing Revision' folder to locate your manuscript file.

Please include the following items when submitting your revised manuscript:

- A rebuttal letter that responds to each point raised by the academic editor and reviewer(s). You should upload this letter as a separate file labeled 'Response to Reviewers'.
- A marked-up copy of your manuscript that highlights changes made to the original version. You should upload this as a separate file labeled 'Revised Manuscript with Track Changes'.
- An unmarked version of your revised paper without tracked changes. You should upload this as a separate file labeled 'Manuscript'.

If you would like to make changes to your financial disclosure, please include your updated statement in your cover letter. Guidelines for resubmitting your figure files are available below the reviewer comments at the end of this letter.

If applicable, we recommend that you deposit your laboratory protocols in protocols.io to enhance the reproducibility of your results. Protocols.io assigns your protocol its own identifier (DOI) so that it can be cited independently in the future. For instructions see: https://journals.plos.org/plosone/s/submission-guidelines#loc-laboratory-protocols. Additionally, PLOS ONE offers an option for publishing peer-reviewed Lab Protocol articles, which describe protocols hosted on protocols.io. Read more information on sharing protocols at https://plos.org/protocols?utm_medium=editorial-email&utm_source=authorletters&utm_campaign=protocols.

We look forward to receiving your revised manuscript.

Kind regards,

Lakshminarayana Chekuri, MD, PhD

Academic Editor

PLOS ONE

Journal requirements:

1. When submitting your revision, we need you to address these additional requirements.

Please ensure that your manuscript meets PLOS ONE's style requirements, including those for file naming. The PLOS ONE style templates can be found at 

https://journals.plos.org/plosone/s/file?id=wjVg/PLOSOne_formatting_sample_main_body.pdf and 

https://journals.plos.org/plosone/s/file?id=ba62/PLOSOne_formatting_sample_title_authors_affiliations.pdf.

2. We note that you have indicated that there are restrictions to data sharing for this study. PLOS only allows data to be available upon request if there are legal or ethical restrictions on sharing data publicly. For more information on unacceptable data access restrictions, please see http://journals.plos.org/plosone/s/data-availability#loc-unacceptable-data-access-restrictions. 

Before we proceed with your manuscript, please address the following prompts:

a) If there are ethical or legal restrictions on sharing a de-identified data set, please explain them in detail (e.g., data contain potentially identifying or sensitive patient information, data are owned by a third-party organization, etc.) and who has imposed them (e.g., a Research Ethics Committee or Institutional Review Board, etc.). Please also provide contact information for a data access committee, ethics committee, or other institutional body to which data requests may be sent.

b) If there are no restrictions, please upload the minimal anonymized data set necessary to replicate your study findings to a stable, public repository and provide us with the relevant URLs, DOIs, or accession numbers. For a list of recommended repositories, please see

https://journals.plos.org/plosone/s/recommended-repositories. You also have the option of uploading the data as Supporting Information files, but we would recommend depositing data directly to a data repository if possible.

We will update your Data Availability statement on your behalf to reflect the information you provide.

3. Please include your full ethics statement in the ‘Methods’ section of your manuscript file. In your statement, please include the full name of the IRB or ethics committee who approved or waived your study, as well as whether or not you obtained informed written or verbal consent. If consent was waived for your study, please include this information in your statement as well. 

Additional Editor Comments:

Thank you for your scholarly contribution to the topic area of Homelessness. I'd also like to thank the authors for choosing PLOS ONE to publish your findings from this study. My comments and comments from three other experts in this topic area are provided below. Please review these comments and I suggest address them and resubmit your manuscript. Your timely response would help this study be published and will make it accessible to interested readers across the world. I look forward to reviewing your revised manuscript. I wish you good luck with your future endeavors.

Editor comments:

I'd recommend adding "Homelessness" as one of the keywords.

The authors' description that this study used a "comprehensive dataset" appears to be somewhat misleading (page 29). For instance, by the authors' own acknowledgement (page 5) "we restricted the study to participants ≥ 18 years with no missing data on key variables." This suggests that only a partial dataset was used for this analysis. On page 5, the authors state that "9,939 participants" were excluded for various reasons. This could have affected the findings in this study. Such limitations need to be acknowledged in the manuscript.

On Page 11, Table 1, socio-economic section the authors present data on income as discrete categories. However, the groups seem to be overlapping. For instance, respondents earning US$ 25000 have a likelihood of being grouped twice. The same pattern is noted in tables 2 and 3 as well. Please address this.

On Page 7, under substance use and health characteristics you describe one of the questions as “In your entire life, have you had at least 1 drink of any kind of alcohol, not counting small tastes or sips?” Please explain the relevance of "at least 1 drink of any kind of alcohol" consumption and its influence on HI. Did the authors hypothesize that all respondents who consumed "at least 1 drink of any kind of alcohol" in their "entire life" to have an alcohol use disorder and there by carried a higher risk for HI? Likewise, I cannot understand the relevance of "LIFETIME" "EVER USAGE" of drugs and its relation to HI. Please acknowledge these as limitations for this study.

Also, for the question "In your LIFETIME, which of the following substances have you ever used?”, please detail what kind of substances the study screened for.

Finally, as some of the reviewers rightly pointed out, the study has significant limitations, and such limitations need to be acknowledged in the manuscript.

[Note: HTML markup is below. Please do not edit.]

Reviewers' comments:

Reviewer's Responses to Questions

**Comments to the Author**

1. Is the manuscript technically sound, and do the data support the conclusions?

The manuscript must describe a technically sound piece of scientific research with data that supports the conclusions. Experiments must have been conducted rigorously, with appropriate controls, replication, and sample sizes. The conclusions must be drawn appropriately based on the data presented. 

Reviewer #1: Partly

Reviewer #2: Yes

Reviewer #3: Yes

**********

2. Has the statistical analysis been performed appropriately and rigorously? 

Reviewer #1: No

Reviewer #2: Yes

Reviewer #3: Yes

**********

3. Have the authors made all data underlying the findings in their manuscript fully available?

The PLOS Data policy requires authors to make all data underlying the findings described in their manuscript fully available without restriction, with rare exception (please refer to the Data Availability Statement in the manuscript PDF file). The data should be provided as part of the manuscript or its supporting information, or deposited to a public repository. For example, in addition to summary statistics, the data points behind means, medians and variance measures should be available. If there are restrictions on publicly sharing data—e.g. participant privacy or use of data from a third party—those must be specified.

Reviewer #1: Yes

Reviewer #2: Yes

Reviewer #3: Yes

**********

4. Is the manuscript presented in an intelligible fashion and written in standard English?

PLOS ONE does not copyedit accepted manuscripts, so the language in submitted articles must be clear, correct, and unambiguous. Any typographical or grammatical errors should be corrected at revision, so please note any specific errors here.

Reviewer #1: Yes

Reviewer #2: Yes

Reviewer #3: Yes

**********

5. Review Comments to the Author

Please use the space provided to explain your answers to the questions above. You may also include additional comments for the author, including concerns about dual publication, research ethics, or publication ethics. (Please upload your review as an attachment if it exceeds 20,000 characters)

Reviewer #1: This manuscript focuses on housing insecurity (HI) and veteran status based on analysis of a large survey dataset. While this promises some interesting and important findings, I found the paper conceptually muddled and unfocused. I’m recommending some major revisions.

First, from the manuscript’s lead sentence, the authors link previous research on HI (which is sparse) with research on homelessness (which is much more plentiful), especially insofar as it involves veterans. Although they are careful to differentiate the two overlapping phenomena, they at least implicitly promise insights into homelessness with this analysis with sentences such as “[u]pdated studies may be needed to maintain a current understanding of homelessness among veterans” (62-63) that it simply never delivers. Nor does it need to as HI is a topic well worth additional study by itself.

Instead of linking HI with homelessness, I urge the authors to consider linking it to homeless prevention, if in no other way that both phenomena involve a population that appears to be at imminent risk of becoming homeless, but most manage to maintain their housing (however precarious) and avoid homelessness. Looked at in a veteran context, the subgroup considered as HI in this paper (persons worried about losing their housing) would be more germane to a constituency served by Supportive Services for Veterans and their Families (SSVF) than by an interim housing program such as Grant and Per Diem (GPD).

Second, the paper never explains why veteran and non-veteran populations could be expected to differ in the prevalence of HI experiences. Beyond the Fargo et al. study cited in the manuscript, there is little evidence to support a higher prevalence of homelessness among non-veterans, and there is also a paradox that I’m sure the authors are aware where veterans should be at lower risk for homelessness due to better economic, employment and educational outcomes, and a more robust social safety net due to the VA. Additionally, given the relatively advanced mean age of the survey respondents, for many of the respondents there has been a time lag spanning multiple decades between their military service and recent HI, which would attenuate associations between the two experiences.

Beyond this, there is little rationale given for examining relationships between HI and the other characteristics that become covariates for the analyses. What results is a study that is basically exploratory, and comes up with an aggregation of characteristics that are associated with HI rather than a clear narrative or framework that these characteristics could fit into. This leads to the first two paragraphs of the discussion section, which I find difficult to find any clear “take-home” (sic) message. Even the study’s main finding, that there was no association found between veteran status and HI experience, is undercut by the authors’ ex post facto equivocations. So why do the study at all?

Additionally, many of the significant associations between covariates and HI experience that the authors highlight are statistically significant due to the large n of the study group, but are arguably non-substantial based on the magnitude of the odds ratio. The authors need to address this.

Finally, the authors gloss over several substantial findings in their discussion of the results that would call into question whether HI experience involved similar dynamics as experiences of homelessness. The most opaque of these is race, in which the authors compare all races to Asian, which makes the racial differences harder to compare and obscures the lack of apparent differences between Black and White races in the odds of experiencing HI. This would be very different from homelessness, where being black almost universally increases the risk for homelessness across many studies. Similarly, the non-significant or extremely modest associations between alcohol and substance use and HI experience runs counter to the relationships generally found between these covariates and homelessness.

Put all of this together, and I really can’t figure out what I should take away from this study.

Reviewer #2: Thank you for the opportunity to review this important research providing a timely, comprehensive account of HI among veterans and non-veterans in the U.S.

One minor revision recommended is that the Introduction briefly describe the difference between housing insecurity (HI) and homelessness for readers who may be unfamiliar with HI. As the authors state: “HI has been identified as a key predictor for homelessness.” A simple contrast could say: Someone who is housing insecure may be unable to pay rent or utilities, may live in substandard housing or overcrowded conditions, or need to move repeatedly. Homelessness, i.e., having no place to live, sleeping on the streets or in shelters, is the ultimate expression of housing insecurity.

Second minor revision is to tighten line spacing in the tables. Also consider eliminating all vertical borders while maintaining only the horizonal borders that separate key variables. These improvements will streamline tables and make them more legible.

Reviewer #3: This manuscript outlines a secondary data analysis of a large survey-based dataset to compare rates and correlates of housing instability in US Veterans and non-Veterans. Overall, I found this paper to be well organized and its topic to make a significant contribution to the broader literature. I have only minor comments as listed below:

INTRODUCTION

* Use of symbols in the opening paragraph is distracting and reduces readability. Please replace symbols (e.g., instead of ">," spell out "more than"; instead of "~," spell out "approximately")

* Line 66 seems to have a typo - "suggesting" should be "suggested"

* Please include conceptualizations/operationalizations of homelessness and housing instability in the Introduction to better orient the reader to the topic of study. These conceptualizations should also serve to highlight the unique contribution of this study to the broader literature given its focus on housing instability rather than homelessness.

METHOD

* Please see comment above regarding use of symbols

* Line 101 suggests participants with missing data were excluded from the sample; however, line 102-103 appears to contradict this by implying that further participants were excluded due to skipping an item about housing instability. Please clarify.

* In describing individual items, it is unclear why some words are provided in all caps.

RESULTS

* What do authors make of the exceptionally high rates of unemployment? Unemployment rates appear particularly at odds with reported annual incomes

* Line 164 reads as if only 3.3% and 7.6% of participants reported any history of alcohol consumption; please reword to reflect that these proportions denied history of alcohol consumption (or instead include statistics for those reporting history of alcohol consumption)

DISCUSSION

* Given the limitations to sampling procedures, I would discourage authors from making claims about prevalence within the discussion (e.g., line 311).

* Can authors speak more to implications of the current study's focus on housing instability rather than homelessness? There is some comparison of results to those of studies focusing on homelessness; however, these are two distinct constructs and additional discussion about implications for housing instability specifically would be helpful.

**********

6. PLOS authors have the option to publish the peer review history of their article (what does this mean?). If published, this will include your full peer review and any attached files.

If you choose “no”, your identity will remain anonymous but your review may still be made public.

**Do you want your identity to be public for this peer review?** For information about this choice, including consent withdrawal, please see our Privacy Policy.

Reviewer #1: **Yes: **Steve Metraux

Reviewer #2: No

Reviewer #3: **Yes: **Emily Edwards

**********

[NOTE: If reviewer comments were submitted as an attachment file, they will be attached to this email and accessible via the submission site. Please log into your account, locate the manuscript record, and check for the action link "View Attachments". If this link does not appear, there are no attachment files.]

While revising your submission, please upload your figure files to the Preflight Analysis and Conversion Engine (PACE) digital diagnostic tool, https://pacev2.apexcovantage.com/. PACE helps ensure that figures meet PLOS requirements. To use PACE, you must first register as a user. Registration is free. Then, login and navigate to the UPLOAD tab, where you will find detailed instructions on how to use the tool. If you encounter any issues or have any questions when using PACE, please email PLOS at figures@plos.org. Please note that Supporting Information files do not need this step.

Attachment Submitted filename: PONE-D-24-25667 REVIEW.docx

# Author comment: Author response to Decision Letter 0

**DOI:** 10.1371/journal.pone.0314339.r002

29 Aug 2024

Additional Editor Comments:

Thank you for your scholarly contribution to the topic area of Homelessness. I'd also like to thank the authors for choosing PLOS ONE to publish your findings from this study. My comments and comments from three other experts in this topic area are provided below. Please review these comments and I suggest address them and resubmit your manuscript. Your timely response would help this study be published and will make it accessible to interested readers across the world. I look forward to reviewing your revised manuscript. I wish you good luck with your future endeavors.

Editor comments:

Comment #1: I'd recommend adding "Homelessness" as one of the keywords.

RESPONSE: We acknowledge the editor’s comment. Accordingly, we have added homelessness as one of the keywords listed after the Abstract.

Comment #2: The authors' description that this study used a "comprehensive dataset" appears to be somewhat misleading (page 29). For instance, by the authors' own acknowledgement (page 5) "we restricted the study to participants ≥ 18 years with no missing data on key variables." This suggests that only a partial dataset was used for this analysis. On page 5, the authors state that "9,939 participants" were excluded for various reasons. This could have affected the findings in this study. Such limitations need to be acknowledged in the manuscript.

RESPONSE: We acknowledge the editor’s comment. Accordingly, we revised a sentence within the last paragraph of the Discussion section by deleting the phrase “comprehensive dataset”, as follows: 

“This study is among few that have used such a large sample to provide a contemporaneous picture of HI rather than homelessness among a racially and ethnically diverse group of veterans and non-veterans in the U.S., while extending previous research on this topic.” 

Furthermore, we added details to the limitations sub-section of the Discussion section in order to address selection bias that may have resulted from the eligibility criteria within the All Of Us Research Program and our study, as follows:

“Fourth, selection bias is likely because of eligibility criteria for participating in the AoU Research Program which restricted the population to individuals ≥ 18 years of age, with the population further restricted based on small cell sizes and availability of data on key variables, including veteran status, HI, and risk/protective characteristics. Non-response to surveys or items within surveys may have specifically resulted in selection bias.” 

Comment #3: On Page 11, Table 1, socio-economic section the authors present data on income as discrete categories. However, the groups seem to be overlapping. For instance, respondents earning US$ 25000 have a likelihood of being grouped twice. The same pattern is noted in tables 2 and 3 as well. Please address this.

RESPONSE: We acknowledge the editor’s comment. To clarify, we are performing secondary analyses of existing data from the All of Us Research Program. The documentation provided online by the All of Us Research Program for the “Household income” item from the “Basics” survey through the Data Browser (View Survey Questions and Answers | All of Us Public Data Browser (researchallofus.org)) suggests that these are response categories were as follows: ‘Annual income: less than 10k’, ‘Annual income: 10k 25k’, ‘Annual income: 25k 35k’, ‘Annual income: 35k 50k’, ‘Annual income: 50k 75k’, ‘Annual income: 75k 100k’, ‘Annual income: 100k 150k’, ‘Annual income: 100k 150k’, ‘Annual income: 150k 200k’, ‘Annual income: more 200k’, ‘Skip’, ‘Prefer Not to Answer’. After removing missing data on this variable, we were able to translate these labels as follows: < 10,000, 10,000-25,000, 25,000-35,000, 35,000-50,000, 50,000-75,000, 75,000-100,000, 100,000-150,000, and > 200,000. Since we are researchers using secondary data from the All Of Us Research Program, we added a limitation within the Discussion section that generally pertains to secondary data use, while highlighting the issue of annual household income categories, as follows:

“First, use of secondary data from the AoU Research Program restricted the scope of risk and protective characteristics evaluated in relation to HI and veteran status, and our ability to collect detailed information on alcohol consumption and drug use, or to re-define overlapping categories for annual household income.”

Comment #4: On Page 7, under substance use and health characteristics you describe one of the questions as “In your entire life, have you had at least 1 drink of any kind of alcohol, not counting small tastes or sips?” Please explain the relevance of "at least 1 drink of any kind of alcohol" consumption and its influence on HI. Did the authors hypothesize that all respondents who consumed "at least 1 drink of any kind of alcohol" in their "entire life" to have an alcohol use disorder and there by carried a higher risk for HI? Likewise, I cannot understand the relevance of "LIFETIME" "EVER USAGE" of drugs and its relation to HI. Please acknowledge these as limitations for this study.

RESPONSE: We acknowledge the editor’s comment. Accordingly, we revised the limitations sub-section within the Discussion section to clarify our choices of study variables to define alcohol consumption and drug use, as well as opportunities for future analyses within the All of Us Research Program:

“Attempting to reduce the issue of reverse causation, we evaluated lifetime rather than current alcohol consumption and drug use in relation to recent experiences with HI. Given the dynamic nature of substance use and the fact that ever use of substances may not necessarily indicate a SUDs, future studies should examine associations between diagnosed SUDs and HI among AoU Research Program participants with available EHR data.”

Also, for the question "In your LIFETIME, which of the following substances have you ever used?”, please detail what kind of substances the study screened for.

RESPONSE: We acknowledge the editor’s comment. Accordingly, we added details regarding drug use, i.e., the types of substances that were screened for within the “Lifestyle” survey of the All of Us Research Program as follows:

“Using the “Lifestyle” survey component we defined any lifetime history of substance use as dichotomous (Yes or No) variables for cigarette smoking (“Have you smoked at least 100 cigarettes in your entire life?”), alcohol consumption (“In your entire life, have you had at least 1 drink of any kind of alcohol, not counting small tastes or sips?”) and drug use (“In your lifetime, which of the following substances have you ever used?”), with drug types categorized as marijuana, hallucinogens, cocaine, methamphetamines, prescription stimulants, sedatives, prescription opioids, street opioids, inhalants, and other drugs. Notably, any lifetime use of a substance, in this context, included even a one-time use”.

Finally, as some of the reviewers rightly pointed out, the study has significant limitations, and such limitations need to be acknowledged in the manuscript.

RESPONSE: We acknowledge the editor’s comments. Accordingly, we made every effort to address and/or acknowledge these study limitations, as indicated by the reviewers.

Please see an acknowledgment of these limitations pertaining to substance use within the Discussion section:

“It is important to note that substance use disorders (SUDs) are a known risk factor for homelessness, but we broadly examined any use of alcohol and drugs in a participant’s lifetime, including even a one-time substance use experience. A such, our findings regarding substance use may not be comparable to those of studies that assessed more problematic substance use or SUDs.”

“Second, the cross-sectional design precludes our ability to draw conclusions pertaining to the temporal relationship between the selected characteristics and HI or between veteran status and HI. Attempting to reduce the issue of reverse causation, we evaluated lifetime rather than current alcohol consumption and drug use in relation to recent experiences with HI. Given the dynamic nature of substance use and the fact that ever use of substances may not necessarily indicate a SUDs, future studies should examine associations between diagnosed SUDs and HI among AoU Research Program participants with available EHR data.”

Reviewer #1: 

Comment #1: This manuscript focuses on housing insecurity (HI) and veteran status based on analysis of a large survey dataset. While this promises some interesting and important findings, I found the paper conceptually muddled and unfocused. I’m recommending some major revisions.

RESPONSE: We acknowledge the reviewer’s comments. Accordingly, we made every effort to focus the paper based on the stated objectives and performed major revisions, as requested.

Comment #2: First, from the manuscript’s lead sentence, the authors link previous research on HI (which is sparse) with research on homelessness (which is much more plentiful), especially insofar as it involves veterans. Although they are careful to differentiate the two overlapping phenomena, they at least implicitly promise insights into homelessness with this analysis with sentences such as “updated studies may be needed to maintain a current understanding of homelessness among veterans” (62-63) that it simply never delivers. Nor does it need to as HI is a topic well worth additional study by itself. Instead of linking HI with homelessness, I urge the authors to consider linking it to homeless prevention, if in no other way that both phenomena involve a population that appears to be at imminent risk of becoming homeless, but most manage to maintain their housing (however precarious) and avoid homelessness. 

RESPONSE: We acknowledge the reviewer’s comments. We made several changes within the Introduction and Discussion sections that clarify the concepts of homelessness and HI, as well as evidence pertaining to these concepts. To emphasize the link between HI and homelessness prevention, we revised the sentence the reviewer is referring to as follows:

“Accordingly, up-to-date studies may be needed to maintain a current understanding of HI for the purpose of homelessness prevention through services provided to veteran and non-veteran populations.”

Comment #3: Looked at in a veteran context, the subgroup considered as HI in this paper (persons worried about losing their housing) would be more germane to a constituency served by Supportive Services for Veterans and their Families (SSVF) than by an interim housing program such as Grant and Per Diem (GPD).

RESPONSE: We acknowledge the reviewer’s comment. Accordingly, we have added a sentence within the Conclusion section to address relevance to VA programs, as shown below:

“Studies comparing veterans at risk for homelessness to those already experiencing homelessness will likely inform VA programs such as the ‘Supportive Services for Veterans and their Families’ and the ‘Grant and Per Diem’ regarding their constituencies.”

Comment #4: Second, the paper never explains why veteran and non-veteran populations could be expected to differ in the prevalence of HI experiences. Beyond the Fargo et al. study cited in the manuscript, there is little evidence to support a higher prevalence of homelessness among non-veterans, and there is also a paradox that I’m sure the authors are aware where veterans should be at lower risk for homelessness due to better economic, employment and educational outcomes, and a more robust social safety net due to the VA. Additionally, given the relatively advanced mean age of the survey respondents, for many of the respondents there has been a time lag spanning multiple decades between their military service and recent HI, which would attenuate associations between the two experiences.

RESPONSE: We acknowledge the reviewer’s comment. Accordingly, we added a sentence within the Introduction section that explains why veterans may differ in terms of HI experiences than their non-veteran counterparts, as follows:

“U.S. veterans constitute a special population that may be at increased risk for HI and homelessness because of detrimental experiences before, during, and after military service, although these risks may paradoxically be mitigated by demographic and socioeconomic characteristics that favor veterans and the availability of social services through the U.S. Department of Veterans Affairs (VA) [9].”

Comment #5: Beyond this, there is little rationale given for examining relationships between HI and the other characteristics that become covariates for the analyses. What results is a study that is basically exploratory, and comes up with an aggregation of characteristics that are associated with HI rather than a clear narrative or framework that these characteristics could fit into. This leads to the first two paragraphs of the discussion section, which I find difficult to find any clear “take-home” (sic) message. Even the study’s main finding, that there was no association found between veteran status and HI experience, is undercut by the authors’ ex post facto equivocations. So why do the study at all?

RESPONSE: We acknowledge the reviewer’s comment. To clarify, this study is exploratory in nature, and was conducted due the scarcity of evidence pertaining to HI. The main purpose of this study is to provide a picture for the epidemiology of HI by examining demographic, socioeconomic, substance use, and health characteristics among veteran and non-veteran populations, as stated within the Introduction section. We believe the association between veteran status and HI is not significant and that is notable (i.e., null results are informative). As such, we revised several sentences within the Introduction section, as follows:

“Recent studies focused on HI and homelessness among veteran and non-veteran populations have identified a wide range of socio-demographic (e.g. sex, age, race/ethnicity, education, income, health insurance, foster care, entitlements, veteran status), behavioral (e.g. smoking status, alcohol consumption, substance use, sexual practices, sensation-seeking, aggression) and clinical (e.g. body mass index, chronic conditions, physical/mental health) correlates for these outcomes[4-6, 21]. Unlike homelessness which is the ultimate expression of HI, evidence pertaining to HI among veterans has been limited, with several studies focused on evaluating HI screening and intervention activities, and fewer of these studies focused on identifying risk/protective factors for HI[6, 11, 22-36]. In this exploratory study, we used data from the All of Us Research Program (AoU) – a unique database funded by the U.S. National Institute of Health – to examine the epidemiology of HI among U.S. veteran and non-veteran adult participants. We aimed to address the following research questions in our analyses: [1] which demographic, socioeconomic, substance use, and health characteristics previously linked to homelessness are associated with HI among veteran and non-veterans? and [2] Is veteran status associated with HI, net of other factors? Study findings are expected to contribute to the current understanding of HI epidemiology by veteran status.”

Furthermore, we revised and combined the first two paragraphs of the Discussion section for the purpose of clarity when it comes to the relationship between veteran status and HI, as shown below:

“HI remains prevalent among both veteran and non-veteran populations in the U.S.[44-46]. Our analysis of cross-sectional data on more than 200,000 veteran and non-veteran participants from AoU found no significant differences in rates of HI over the past 6 months between veterans and non-veterans after controlling for various individual-level characteristics. Whereas veterans represent nearly 10% of homeless people in the U.S. [5], it may be important to consider that veterans may have unique vulnerabilities that predispose them to homelessness and HI, including those stemming from a history of combat exposure[9] or military sexual trauma[47], although these vulnerabilities may be counteracted by unique protective factors, such as access to health care and case management supports t

Attachment Submitted filename: AOU_MAN_1_HI_PREDICTORS_DRAFT10_Response to Reviewers.docx

# Peer review: Decision Letter 1

**DOI:** 10.1371/journal.pone.0314339.r003

**Authors:** Lakshminarayana Chekuri

27 Sep 2024

PONE-D-24-25667R1Correlates of six-month housing instability among U.S. adults by veteran status: Data from the All of Us Research ProgramPLOS ONE

Dear Dr. Tsai,

Thank you for submitting your manuscript to PLOS ONE. After careful consideration, we feel that it has merit but does not fully meet PLOS ONE’s publication criteria as it currently stands. Therefore, we invite you to submit a revised version of the manuscript that addresses the points raised during the review process.

Please submit your revised manuscript by Nov 11 2024 11:59PM. If you will need more time than this to complete your revisions, please reply to this message or contact the journal office at plosone@plos.org. When you're ready to submit your revision, log on to https://www.editorialmanager.com/pone/ and select the 'Submissions Needing Revision' folder to locate your manuscript file.

Please include the following items when submitting your revised manuscript:

- A rebuttal letter that responds to each point raised by the academic editor and reviewer(s). You should upload this letter as a separate file labeled 'Response to Reviewers'.
- A marked-up copy of your manuscript that highlights changes made to the original version. You should upload this as a separate file labeled 'Revised Manuscript with Track Changes'.
- An unmarked version of your revised paper without tracked changes. You should upload this as a separate file labeled 'Manuscript'.

If you would like to make changes to your financial disclosure, please include your updated statement in your cover letter. Guidelines for resubmitting your figure files are available below the reviewer comments at the end of this letter.

If applicable, we recommend that you deposit your laboratory protocols in protocols.io to enhance the reproducibility of your results. Protocols.io assigns your protocol its own identifier (DOI) so that it can be cited independently in the future. For instructions see: https://journals.plos.org/plosone/s/submission-guidelines#loc-laboratory-protocols. Additionally, PLOS ONE offers an option for publishing peer-reviewed Lab Protocol articles, which describe protocols hosted on protocols.io. Read more information on sharing protocols at https://plos.org/protocols?utm_medium=editorial-email&utm_source=authorletters&utm_campaign=protocols.

We look forward to receiving your revised manuscript.

Kind regards,

Lakshminarayana Chekuri, MD, PhD

Academic Editor

PLOS ONE

Journal Requirements:

Please review your reference list to ensure that it is complete and correct. If you have cited papers that have been retracted, please include the rationale for doing so in the manuscript text, or remove these references and replace them with relevant current references. Any changes to the reference list should be mentioned in the rebuttal letter that accompanies your revised manuscript. If you need to cite a retracted article, indicate the article’s retracted status in the References list and also include a citation and full reference for the retraction notice.

Additional Editor Comments:

Thank you for your scholarly contribution to the topic area of Homelessness. I'd also like to thank the authors for choosing PLOS ONE to publish your findings from this study. I believe this manuscript is getting close to acceptance for a publication. My comments and comments from reviewer 1 are provided below. Please review these comments and I suggest address them and resubmit your manuscript. Your timely response would help this study be published and accessible to interested readers across the world. I look forward to reviewing your revised manuscript. I wish you good luck with your future endeavors.

Editor comments:

line 53 – Please consider adding "in the U.S." at the end of the sentence

lines 89-90: You state "Unlike homelessness which is the ultimate expression of HI....", please provide a citation for your assertion "homelessness .... is the ultimate expression of HI".

Table 2: please consider adding a statement that states percentages were rounded to first decimal.

I could not find "Figure 1" that is referenced across the manuscript. Please provide this for my review.

In the "materials and methods" section (line 100): If available, please provide response rate and total number of surveys sent out.

On line 290: Please correct typo "A such" to "As such"

The abbreviation "VA" was used throughout the manuscript. I could not find the full expansion of this abbreviation in the manuscript. I'd recommend provide a full expansion of this abbreviation at the first instance.

[Note: HTML markup is below. Please do not edit.]

Reviewers' comments:

Reviewer's Responses to Questions

**Comments to the Author**

1. If the authors have adequately addressed your comments raised in a previous round of review and you feel that this manuscript is now acceptable for publication, you may indicate that here to bypass the “Comments to the Author” section, enter your conflict of interest statement in the “Confidential to Editor” section, and submit your "Accept" recommendation.

Reviewer #1: (No Response)

Reviewer #2: All comments have been addressed

**********

2. Is the manuscript technically sound, and do the data support the conclusions?

The manuscript must describe a technically sound piece of scientific research with data that supports the conclusions. Experiments must have been conducted rigorously, with appropriate controls, replication, and sample sizes. The conclusions must be drawn appropriately based on the data presented. 

Reviewer #1: Partly

Reviewer #2: Yes

**********

3. Has the statistical analysis been performed appropriately and rigorously? 

Reviewer #1: Yes

Reviewer #2: Yes

**********

4. Have the authors made all data underlying the findings in their manuscript fully available?

The PLOS Data policy requires authors to make all data underlying the findings described in their manuscript fully available without restriction, with rare exception (please refer to the Data Availability Statement in the manuscript PDF file). The data should be provided as part of the manuscript or its supporting information, or deposited to a public repository. For example, in addition to summary statistics, the data points behind means, medians and variance measures should be available. If there are restrictions on publicly sharing data—e.g. participant privacy or use of data from a third party—those must be specified.

Reviewer #1: (No Response)

Reviewer #2: Yes

**********

5. Is the manuscript presented in an intelligible fashion and written in standard English?

PLOS ONE does not copyedit accepted manuscripts, so the language in submitted articles must be clear, correct, and unambiguous. Any typographical or grammatical errors should be corrected at revision, so please note any specific errors here.

Reviewer #1: Yes

Reviewer #2: Yes

**********

6. Review Comments to the Author

Please use the space provided to explain your answers to the questions above. You may also include additional comments for the author, including concerns about dual publication, research ethics, or publication ethics. (Please upload your review as an attachment if it exceeds 20,000 characters)

Reviewer #1: I appreciate the authors’ attentiveness to my concerns and comments regarding the original version of their manuscript. In responding to their revised text, I’ll group my comments by those pertaining to the introductory text and then to the text in the discussion/conclusion sections.

Regarding the introductory text, I still have problems with how the authors frame the study. When I commented, in my initial review, that the authors frame this study in the context of housing insecurity and homelessness prevention instead of in terms of actual homelessness (which this study really doesn’t address), I meant the authors do more than just rephrase a few passages and insert the term “housing insecurity” into a few places while keeping the initial references and keeping the structure of the introduction largely unchanged.

On reading the revised manuscript, the disconnect between HI, as the authors look at it and what the data contains, and homelessness is more stark. This is not a homelessness paper at all, as the large majority of those who express HI, such as is examined in this manuscript, will never go on to experience homelessness and the data for this study has no indicator for actual homelessness. Thus, for example, the numbers the authors cite about the incidence of veteran homelessness are largely irrelevant to the paper. The authors’ attempt to connect the two to connect the two gets even more tenuous when their stated definition of HI, as presented at the start of the manuscript, conflicts with their working definition of homelessness as “the ultimate expression of homelessness” (see line 98-99). Finally, while there is not a large literature on HI, there is a literature on this that includes research both on housing instability (see for example AE Montgomery and colleagues’ work with the VA housing screener) and on homeless prevention that directly informs what the authors are looking at here and needs to be included to set the context for the study at hand.

The sentence that the authors inserted (“U.S. veterans constitute…”) in response to what they designated as my “comment #4” would do well as the lead sentence in the manuscript rather than be buried in the middle of a long first paragraph.

Moving to the comments pertaining to the material in the Discussion and Conclusion sections, the authors main finding is that they fail to find a significant difference in the risk for HI between veterans and nonveterans (and the unadjusted difference actually has significantly lower rates of HI for veterans). In explaining this, they speculate on a balancing out of risk factors and protective factors among veterans. But in doing this they fail to consider the simpler option, failing to reject the implicit null hypothesis that there is no evidence of any inherent veteran/non-veteran difference in risk for HI. This latter explanation is the more parsimonious one, and in justifying their alternative explanation on offsetting protective and risk factors, they use research on homelessness for support where there is no evidence to equate risk for HI with risk of homelessness, and also mention a study on risk factors for HI among veterans that, if such a study in fact exists, they would need to cite that study rather than the review article they currently cite to back up their assertion that “unique vulnerabilities” may predispose veterans to HI.

Beyond that, the conclusions they make are, as they now acknowledge, exploratory and focus on risk for HI more generally (i.e., among both veteran and nonveteran populations). I appreciate their attentiveness to my comments about findings related to race, substance use, and significance and effect size, however, the main message I get from the discussion is that there are an overwhelming number of factors that indicate some degree of significant risk for homelessness without a clear framework for organizing the risk or much of how to translate these risks to more applied settings.

In summary, to respond to my continued concerns would still require major revisions along the lines of the issues that I initially raised in my first review.

Reviewer #2: Thank you for addressing this reviewer's comments. The revised manuscript is technically sound, the data support the conclusions, and the writing is articulate. The the statistical analysis been performed appropriately and rigorously. Underlying findings are made available.

**********

7. PLOS authors have the option to publish the peer review history of their article (what does this mean?). If published, this will include your full peer review and any attached files.

If you choose “no”, your identity will remain anonymous but your review may still be made public.

**Do you want your identity to be public for this peer review?** For information about this choice, including consent withdrawal, please see our Privacy Policy.

Reviewer #1: **Yes: **Stephen Metraux

Reviewer #2: No

**********

[NOTE: If reviewer comments were submitted as an attachment file, they will be attached to this email and accessible via the submission site. Please log into your account, locate the manuscript record, and check for the action link "View Attachments". If this link does not appear, there are no attachment files.]

While revising your submission, please upload your figure files to the Preflight Analysis and Conversion Engine (PACE) digital diagnostic tool, https://pacev2.apexcovantage.com/. PACE helps ensure that figures meet PLOS requirements. To use PACE, you must first register as a user. Registration is free. Then, login and navigate to the UPLOAD tab, where you will find detailed instructions on how to use the tool. If you encounter any issues or have any questions when using PACE, please email PLOS at figures@plos.org. Please note that Supporting Information files do not need this step.

# Author comment: Author response to Decision Letter 1

**DOI:** 10.1371/journal.pone.0314339.r004

3 Oct 2024

Journal Requirements:

Please review your reference list to ensure that it is complete and correct. If you have cited papers that have been retracted, please include the rationale for doing so in the manuscript text, or remove these references and replace them with relevant current references. Any changes to the reference list should be mentioned in the rebuttal letter that accompanies your revised manuscript. If you need to cite a retracted article, indicate the article’s retracted status in the References list and also include a citation and full reference for the retraction notice.

RESPONSE: In the process of revising this manuscript, we reviewed the reference list to ensure that it is complete and correct. Furthermore, we ensured that none of the references cited have been retracted. Finally, we would like to indicate that several references were either added or deleted from the reference list based on comments provided by the editor and reviewers. 

Additional Editor Comments:

Thank you for your scholarly contribution to the topic area of Homelessness. I'd also like to thank the authors for choosing PLOS ONE to publish your findings from this study. I believe this manuscript is getting close to acceptance for a publication. My comments and comments from reviewer 1 are provided below. Please review these comments and I suggest address them and resubmit your manuscript. Your timely response would help this study be published and accessible to interested readers across the world. I look forward to reviewing your revised manuscript. I wish you good luck with your future endeavors.

Editor comments:

line 53 – Please consider adding "in the U.S." at the end of the sentence

RESPONSE: We agree with the editorial comment. Accordingly, we added “in the U.S.” at the end of the sentence on line 53, as follows:

“In 2023, it was estimated that more than 600,000 individuals were homeless on a given night in the U.S. [10].”

lines 89-90: You state "Unlike homelessness which is the ultimate expression of HI....", please provide a citation for your assertion "homelessness .... is the ultimate expression of HI".

RESPONSE: We acknowledge the editorial comment. Accordingly, we provided a citation for the assertion that “homelessness .... is the ultimate expression of HI” on line 89-90, and revised the sentence as follows: 

“HI is an important social determinant of health affecting adults in the U.S. [4-7], and homelessness, defined as lack of stable, safe, and functioning housing, is a severe manifestation of HI [8, 9].”

The reference is shown below.

https://www.ncbi.nlm.nih.gov/pmc/articles/PMC6445694/

https://health.gov/healthypeople/priority-areas/social-determinants-health/literature-summaries/housing-instability#:~:text=Evictions%20that%20go%20through%20the,8%2C20

Table 2: please consider adding a statement that states percentages were rounded to first decimal.

RESPONSE: We acknowledge the editorial comment. Accordingly, we added the following statement as a footnote to Table 2:

“Percentages were rounded to one decimal place”

I could not find "Figure 1" that is referenced across the manuscript. Please provide this for my review.

RESPONSE: We acknowledge the editorial comment. In the revised version of the manuscript, we made sure to attach Figure 1 to the submission for review.

In the "materials and methods" section (line 100): If available, please provide response rate and total number of surveys sent out.

RESPONSE: We acknowledge the editorial comment. We would like to clarify that, unlike nationally representative surveys, the All of Us Research Program enrolled subjects on a voluntary basis. Also, the All of Us surveys that we analyzed in this study were required rather than optional surveys. Therefore, response rates for these surveys was high. Please see below the revised sentence within the Methods section:

“Self-reported data were obtained on a subset of 397,191 participants who completed the three non-optional surveys, namely, “The Basics”, “Lifestyle”, and “Overall Health”, with a response rate of 99.8% among study-eligible participants enrolled in the AoU program.”.

On line 290: Please correct typo "A such" to "As such"

RESPONSE: We acknowledge the editorial comment. Accordingly, we revised “A such” to “As such” on line 290.

The abbreviation "VA" was used throughout the manuscript. I could not find the full expansion of this abbreviation in the manuscript. I'd recommend provide a full expansion of this abbreviation at the first instance.

RESPONSE: We acknowledge the editorial comment. Please note that we had provided a full expansion of “VA” abbreviation at the first instance of use, as follows:

“U.S. veterans constitute a special population that may be at increased risk for HI because of detrimental experiences before, during, and after military service, although these risks may be partly mitigated by demographic and socioeconomic characteristics that favor veterans and the availability of health and social services through the U.S. Department of Veterans Affairs (VA) [3].”

6. Review Comments to the Author

Please use the space provided to explain your answers to the questions above. You may also include additional comments for the author, including concerns about dual publication, research ethics, or publication ethics. (Please upload your review as an attachment if it exceeds 20,000 characters)

Reviewer #1: I appreciate the authors’ attentiveness to my concerns and comments regarding the original version of their manuscript. In responding to their revised text, I’ll group my comments by those pertaining to the introductory text and then to the text in the discussion/conclusion sections.

Regarding the introductory text, I still have problems with how the authors frame the study. When I commented, in my initial review, that the authors frame this study in the context of housing insecurity and homelessness prevention instead of in terms of actual homelessness (which this study really doesn’t address), I meant the authors do more than just rephrase a few passages and insert the term “housing insecurity” into a few places while keeping the initial references and keeping the structure of the introduction largely unchanged.

On reading the revised manuscript, the disconnect between HI, as the authors look at it and what the data contains, and homelessness is more stark. This is not a homelessness paper at all, as the large majority of those who express HI, such as is examined in this manuscript, will never go on to experience homelessness and the data for this study has no indicator for actual homelessness. Thus, for example, the numbers the authors cite about the incidence of veteran homelessness are largely irrelevant to the paper. The authors’ attempt to connect the two gets even more tenuous when their stated definition of HI, as presented at the start of the manuscript, conflicts with their working definition of homelessness as “the ultimate expression of homelessness” (see line 98-99). Finally, while there is not a large literature on HI, there is a literature on this that includes research both on housing instability (see for example AE Montgomery and colleagues’ work with the VA housing screener) and on homeless prevention that directly informs what the authors are looking at here and needs to be included to set the context for the study at hand.

RESPONSE: We thank the reviewer for the constructive feedback on the revised manuscript. Accordingly, we re-wrote the introduction, discussion, and concluding sections to address the reviewer’s comments. Specifically, the focus of these sections is no longer on actual homelessness, but rather on housing instability and homelessness prevention. 

We agree with the reviewer that there is a disconnect between housing instability and actual homelessness, since that majority of individuals who experience housing instability will never experience homelessness. Furthermore, we agree that the All Of Us Research Program data cannot be used to examine actual homelessness, and that the definition provided regarding housing instability conflicts with that of homelessness. As such, we revised the Introduction to focus more on housing instability and homelessness prevention, and to better provide context for this study. Please note that we have cited work related to housing instability in the Introduction section, including work by Montgomery and colleagues, and also presented the most relevant work focused on housing instability in the Discussion section. Please see track-changes within the Introduction and 

Discussion sections.

The sentence that the authors inserted (“U.S. veterans constitute…”) in response to what they designated as my “comment #4” would do well as the lead sentence in the manuscript rather than be buried in the middle of a long first paragraph.

RESPONSE: We acknowledge the reviewer’s comment. In the revised version of the manuscript, the sentence mentioned by the reviewer is the second sentence of the first paragraph of the Introduction. 

“U.S. veterans constitute a special population that may be at increased risk for HI because of detrimental experiences before, during, and after military service, although these risks may be partly mitigated by demographic and socioeconomic characteristics that favor veterans and the availability of health and social services through the U.S. Department of Veterans Affairs (VA) [3].”

Moving to the comments pertaining to the material in the Discussion and Conclusion sections, the authors main finding is that they fail to find a significant difference in the risk for HI between veterans and nonveterans (and the unadjusted difference actually has significantly lower rates of HI for veterans). In explaining this, they speculate on a balancing out of risk factors and protective factors among veterans. But in doing this they fail to consider the simpler option, failing to reject the implicit null hypothesis that there is no evidence of any inherent veteran/non-veteran difference in risk for HI. This latter explanation is the more parsimonious one, and in justifying their alternative explanation on offsetting protective and risk factors, they use research on homelessness for support where there is no evidence to equate risk for HI with risk of homelessness, and also mention a study on risk factors for HI among veterans that, if such a study in fact exists, they would need to cite that study rather than the review article they currently cite to back up their assertion that “unique vulnerabilities” may predispose veterans to HI.

RESPONSE: We agree with the reviewer that emphasis should be placed on failing to reject the null hypothesis of a difference in the prevalence of housing instability between veterans and non-veterans, after controlling for confounders, rather than emphasize the balancing out of risk factors and protective factors among veterans which can also lead to this finding. In the revised version of the Discussion section, we wrote:

“Failure to reject the null hypothesis might suggest no inherent differences between veteran and non-veteran populations in terms of the burden of HI.” 

Furthermore, we provided further literature that is focused on housing instability rather than homelessness, including the study on risk factors for HI among veterans, which is more relevant than the review article on homelessness, which was moved to another paragraph within the Discussion section. Please see track-changes within the paragraph:

“Correlates of HI have not been adequately examined in the general population of U.S. adults and have not been compared according to veteran status. Study findings were consistent with some, but not all, of the existing studies that evaluated correlates of HI among U.S. veterans. One study aimed to develop predictive models of HI and homelessness based on electronic medical record data from 5.8 million veterans who responded to the VA's Homelessness Screening Clinical Reminder, indicating that random forests models were more sensitive in predicting HI and homelessness than logistic regression, but less specific [59]. Another study involving 4,633,069 U.S. veterans found that sociodemographic and health service use variables were positively related to recent HI, with drug and opioid use disorders identified as key predictors of recent HI, thereby informing preventive interventions targeting new episodes of HI [60]. A study using VA data revealed that military sexual trauma, VA benefits inaccessibility, and single or divorced marital status were significant risk factors for HI among women veterans, aligning with a theoretical model emphasizing the importance of traumatic events and isolation, and suggesting new supportive interventions can mitigate their impact [61]. Using data on 38,633 post-9/11 U.S. veterans, a study found that one-third reported food and/or housing instability (FHI), with greater frequency among women and post-service [28]. Furthermore, FHI was associated with adverse childhood experiences, being enlisted, homelessness, depression, low social support, living with seriously ill/disabled persons, and living in dangerous neighborhoods [28]. Posttraumatic stress disorder, cholesterol level, hypertension, and illegal/street drug use were significant correlates of FHI among men, whereas morbid obesity and diabetes were significant correlates of FHI among women [28].” 

Beyond that, the conclusions they make are, as they now acknowledge, exploratory and focus on risk for HI more generally (i.e., among both veteran and nonveteran populations). I appreciate their attentiveness to my comments about findings related to race, substance use, and significance and effect size, however, the main message I get from the discussion is that there are an overwhelming number of factors that indicate some degree of significant risk for homelessness without a clear framework for organizing the risk or much of how to translate these risks to more applied settings.

RESPONSE: We acknowledge the reviewer’s comment. Accordingly, the concluding paragraph was revised to emphasize modifiable and non-modifiable correlates of housing instability for the purpose of homelessness prevention among veteran and non-veteran populations. We emphasized the multifactorial nature of housing instability, and provided practical implications to study findings.

“Furthermore, the burden of HI was shown to be multifactorial in nature. Certain demographic, socioeconomic, substance use, and health characteristics were associated with HI, with some of these modifiable and non-modifiable characteristics (e.g. race, employment, alcohol consumption, drug use) being more salient to non-veterans versus veterans, thereby informing the planning, implementation, and evaluation of evidence-based interventions aimed at addressing HI among veteran and non-veteran populations.”

In summary, to respond to my continued concerns would still require major revisions along the lines of the issues that I initially raised in my first review.

RESPONSE: We acknowledge the reviewer’s comments. Accordingly, major revisions were made to the manuscript, taking into consideration the comments provided by the reviewer at first review.

Reviewer #2: Thank you for addressing this reviewer's comments. The revised manuscript is technically sound, the data support the conclusions, and the writing is articulate. The statistical analysis been performed appropriately and rigorously. Underlying findings are made available.

RESPONSE: We thank the reviewer for the positive feedback on the revised manuscript.

Attachment Submitted filename: PONE_R&R_R1_093024.docx

# Editor report: Decision Letter 2

**DOI:** 10.1371/journal.pone.0314339.r005

**Authors:** Lakshminarayana Chekuri

5 Nov 2024

PONE-D-24-25667R2Correlates of six-month housing instability among U.S. adults by veteran status: Data from the All of Us Research ProgramPLOS ONE

Dear Dr. Tsai,

Thank you for submitting your manuscript to PLOS ONE. After careful consideration, we feel that it has merit but does not fully meet PLOS ONE’s publication criteria as it currently stands. Therefore, we invite you to submit a revised version of the manuscript that addresses the points raised during the review process.

Please submit your revised manuscript by Dec 20 2024 11:59PM. If you will need more time than this to complete your revisions, please reply to this message or contact the journal office at plosone@plos.org. When you're ready to submit your revision, log on to https://www.editorialmanager.com/pone/ and select the 'Submissions Needing Revision' folder to locate your manuscript file.

Please include the following items when submitting your revised manuscript:

- A rebuttal letter that responds to each point raised by the academic editor and reviewer(s). You should upload this letter as a separate file labeled 'Response to Reviewers'.
- A marked-up copy of your manuscript that highlights changes made to the original version. You should upload this as a separate file labeled 'Revised Manuscript with Track Changes'.
- An unmarked version of your revised paper without tracked changes. You should upload this as a separate file labeled 'Manuscript'.

If you would like to make changes to your financial disclosure, please include your updated statement in your cover letter. Guidelines for resubmitting your figure files are available below the reviewer comments at the end of this letter.

If applicable, we recommend that you deposit your laboratory protocols in protocols.io to enhance the reproducibility of your results. Protocols.io assigns your protocol its own identifier (DOI) so that it can be cited independently in the future. For instructions see: https://journals.plos.org/plosone/s/submission-guidelines#loc-laboratory-protocols. Additionally, PLOS ONE offers an option for publishing peer-reviewed Lab Protocol articles, which describe protocols hosted on protocols.io. Read more information on sharing protocols at https://plos.org/protocols?utm_medium=editorial-email&utm_source=authorletters&utm_campaign=protocols.

We look forward to receiving your revised manuscript.

Kind regards,

Lakshminarayana Chekuri, MD, PhD

Academic Editor

PLOS ONE

Journal Requirements:

Please review your reference list to ensure that it is complete and correct. If you have cited papers that have been retracted, please include the rationale for doing so in the manuscript text, or remove these references and replace them with relevant current references. Any changes to the reference list should be mentioned in the rebuttal letter that accompanies your revised manuscript. If you need to cite a retracted article, indicate the article’s retracted status in the References list and also include a citation and full reference for the retraction notice.

Please see attached file.

[Note: HTML markup is below. Please do not edit.]

Reviewers' comments: None

[NOTE: If reviewer comments were submitted as an attachment file, they will be attached to this email and accessible via the submission site. Please log into your account, locate the manuscript record, and check for the action link "View Attachments". If this link does not appear, there are no attachment files.]

While revising your submission, please upload your figure files to the Preflight Analysis and Conversion Engine (PACE) digital diagnostic tool, https://pacev2.apexcovantage.com/. PACE helps ensure that figures meet PLOS requirements. To use PACE, you must first register as a user. Registration is free. Then, login and navigate to the UPLOAD tab, where you will find detailed instructions on how to use the tool. If you encounter any issues or have any questions when using PACE, please email PLOS at figures@plos.org. Please note that Supporting Information files do not need this step.

Attachment Submitted filename: Editor comments 11-03-24 PONE-D-24-25667.docx

# Author comment: Author response to Decision Letter 2

**DOI:** 10.1371/journal.pone.0314339.r006

6 Nov 2024

Dear Dr. Chekuri and Reviewers, 

Thank you for the opportunity to revise and resubmit our manuscript. As requested, we have now used the PACE digital diagnostic tool to ensure that Figure 1 meets PLOS requirements. Below, we detail other changes we have made and appreciate the comments we received. We hope you can now consider the manuscript for publication. Thank you very much.

Editor – suggested changes:

Line 1: Consider adding "Exploratory study of" Data from ..... in the title.

RESPONSE: Done.

Line 25, Consider adding "(U.S)" at the end of this sentence. I see that you used "U.S" to reflect United States from here onwards.

RESPONSE: Done.

Line 80: consider changing "chronic conditions" to "chronic health conditions"

RESPONSE: Done.

Line 102, expand "EHR" and abbreviate in parenthesis, since this is the first time "EHR" is being used in the manuscript.

RESPONSE: Done

Line 156, consider rephrasing "Using the “Lifestyle” survey component we defined any …..”

to "Using the “Lifestyle” survey component we defined assessed any…..” 

RESPONSE: Done.

Line 285: Consider rephrasing the following statement: "Our findings suggest distinct racial disparities between those who experience homelessness and those who report being at risk of experiencing homelessness based on HI." The contents of your current statement “……and those who report being at risk of experiencing homelessness based on HI” appear a bit misleading since your study did not find a relationship between HI and homelessness.

I'd recommend as follows: "Our findings suggest distinct racial disparities between those studies that reported experiences of homeless individuals and our study participants who perceived HI.”

RESPONSE: Done.

Line 366: consider changing "yielding" to "yielded"

RESPONSE: Done.

Line 375: Since this is a concluding statement, I'd recommend consider deleting "substance use" from this sentence. I am recommending this because your study's assessment of substance use has serious limitations, as you rightly acknowledged.

RESPONSE: Done.

Attachment Submitted filename: Response_to_Editorial_and_Reviewers_Comments_PONE_110624.docx

# Editor report: Decision Letter 3

**DOI:** 10.1371/journal.pone.0314339.r007

**Authors:** Lakshminarayana Chekuri

11 Nov 2024

Correlates of six-month housing instability among U.S. adults by veteran status: Exploratory study using data from the All of Us Program

PONE-D-24-25667R3

Dear Dr. Tsai,

We’re pleased to inform you that your manuscript has been judged scientifically suitable for publication and will be formally accepted for publication once it meets all outstanding technical requirements.

Within one week, you’ll receive an e-mail detailing the required amendments. When these have been addressed, you’ll receive a formal acceptance letter and your manuscript will be scheduled for publication.

An invoice will be generated when your article is formally accepted. Please note, if your institution has a publishing partnership with PLOS and your article meets the relevant criteria, all or part of your publication costs will be covered. Please make sure your user information is up-to-date by logging into Editorial Manager at Editorial Manager® and clicking the ‘Update My Information' link at the top of the page. If you have any questions relating to publication charges, please contact our Author Billing department directly at authorbilling@plos.org.

If your institution or institutions have a press office, please notify them about your upcoming paper to help maximize its impact. If they’ll be preparing press materials, please inform our press team as soon as possible -- no later than 48 hours after receiving the formal acceptance. Your manuscript will remain under strict press embargo until 2 pm Eastern Time on the date of publication. For more information, please contact onepress@plos.org.

Kind regards,

Lakshminarayana Chekuri, MD, PhD

Academic Editor

PLOS ONE

Additional Editor Comments (optional):

Reviewers' comments:

# Editor report: Acceptance letter

**DOI:** 10.1371/journal.pone.0314339.r008

**Authors:** Lakshminarayana Chekuri

13 Nov 2024

PONE-D-24-25667R3 

PLOS ONE

Dear Dr. Tsai, 

I'm pleased to inform you that your manuscript has been deemed suitable for publication in PLOS ONE. Congratulations! Your manuscript is now being handed over to our production team.

At this stage, our production department will prepare your paper for publication. This includes ensuring the following:

* All references, tables, and figures are properly cited

* All relevant supporting information is included in the manuscript submission,

* There are no issues that prevent the paper from being properly typeset

If revisions are needed, the production department will contact you directly to resolve them. If no revisions are needed, you will receive an email when the publication date has been set. At this time, we do not offer pre-publication proofs to authors during production of the accepted work. Please keep in mind that we are working through a large volume of accepted articles, so please give us a few weeks to review your paper and let you know the next and final steps. 

Lastly, if your institution or institutions have a press office, please let them know about your upcoming paper now to help maximize its impact. If they'll be preparing press materials, please inform our press team within the next 48 hours. Your manuscript will remain under strict press embargo until 2 pm Eastern Time on the date of publication. For more information, please contact onepress@plos.org.

If we can help with anything else, please email us at customercare@plos.org.

Thank you for submitting your work to PLOS ONE and supporting open access. 

Kind regards, 

PLOS ONE Editorial Office Staff

on behalf of

Dr. Lakshminarayana Chekuri 

Academic Editor

PLOS ONE
